# Master equation simulation analysis of immunostained Bicoid morphogen gradient

**DOI:** 10.1186/1752-0509-1-52

**Published:** 2007-11-16

**Authors:** Yu Feng Wu, Ekaterina Myasnikova, John Reinitz

**Affiliations:** 1 Department of Applied Mathematics and Statistics, and Center for Developmental Genetics, Stony Brook University, Stony Brook, NY 11794-3600, USA; 2 Department of Computational Biology, Center of Advanced Studies, St.Petersburg State Polytechnic University, St.Petersburg, 195251, Russia

## Abstract

**Background:**

The concentration gradient of Bicoid protein which determines the developmental pathways in early *Drosophila *embryo is the best characterized morphogen gradient at the molecular level. Because different developmental fates can be elicited by different concentrations of Bicoid, it is important to probe the limits of this specification by analyzing intrinsic fluctuations of the Bicoid gradient arising from small molecular number. Stochastic simulations can be applied to further the understanding of the dynamics of Bicoid morphogen gradient formation at the molecular number level, and determine the source of the nucleus-to-nucleus expression variation (noise) observed in the Bicoid gradient.

**Results:**

We compared quantitative observations of Bicoid levels in immunostained *Drosophila *embryos with a spatially extended Master Equation model which represents diffusion, decay, and anterior synthesis. We show that the intrinsic noise of an autonomous reaction-diffusion gradient is Poisson distributed. We demonstrate how experimental noise can be identified in the logarithm domain from single embryo analysis, and then separated from intrinsic noise in the normalized variance domain of an ensemble statistical analysis. We show how measurement sensitivity affects our observations, and how small amounts of rescaling noise can perturb the noise strength (Fano factor) observed. We demonstrate that the biological noise level in data can serve as a physical constraint for restricting the model's parameter space, and for predicting the Bicoid molecular number and variation range. An estimate based on a low variance ensemble of embryos suggests that the steady-state Bicoid molecular number in a nucleus should be larger than 300 in the middle of the embryo, and hence the gradient should extend to the posterior end of the embryo, beyond the previously assumed background limit. We exhibit the predicted molecular number gradient together with measurement effects, and make a comparison between conditions of higher and lower variance respectively.

**Conclusion:**

Quantitative comparison of Master Equation simulations with immunostained data enabled us to determine narrow ranges for key biophysical parameters, which for this system can be independently validated. Intrinsic noise is clearly detectable as well, although the staining process introduces certain limits in resolution.

## Background

Recently considerable attention has been given to the characterization and understanding of intrinsic molecular noise in biological systems [1-8]. Nearly all of these studies were performed using *in vivo *fluorescent reporters in single cell systems. In multicellular organisms, however, most quantitative gene expression data are obtained from fixed tissues. Examples of such data for the *Drosophila *segmentation system are contained in the FlyEx database, which provides spatiotemporal data on the expression of developmental segmentation genes [9]. These data on protein expression levels are at cellular resolution and were obtained by means of immunofluorescence histochemistry and confocal scanning microscopy. At a large spatial scale, expression levels in these embryos form expression domains characteristic for each gene, but smaller fluctuations in expression levels between adjacent nuclei appear random. In this paper, we investigate the question of whether these fluctuations are a consequence of intrinsic molecular noise or stem from some type of measurement uncertainty. These alternatives are, of course, not mutually exclusive.

A complicating factor in separating the above alternatives is that each one involves an unknown chemical mechanism. Intrinsic noise will have a major contribution from fluctuations in the rate of initiation of transcription, but the chemical mechanisms underlying this process in multicellular organisms are very poorly understood. Measurement uncertainty can stem from chemical causes such as fluctuations in the number of primary and secondary antibody molecules which bind to proteins in the fixed embryo. The chemistry of this process is also very poorly understood. If observations could be made on a process whose fluctuation properties could be reliably predicted by a numerical model, comparison of the predicted fluctuations with those observed will provide critical information for distinguishing whether observed nucleus-to-nucleus variations are a consequence of intrinsic biological noise or merely fluctuations arising from the staining procedure.

An excellent candidate for a process with predictable fluctuation properties is one that involves only diffusion and first order decay. There is good evidence that the formation of the protein gradient of the morphogen Bicoid (Bcd) takes place by means of these two processes. Bcd protein is distributed in an exponential profile along the anterior-posterior (A-P) axis with higher concentrations towards the anterior [10,11]. This gradient forms by translation of maternally deposited mRNA at the anterior pole of the embryo, and the synthesized protein spreads through the syncytial embryo by diffusion accompanied by decay [12]. The observed exponential profile corresponds to a solution of Fick's equation for a substance undergoing first order decay and diffusing from a point source in one dimension, and hence it is reasonable to suppose that the reaction-diffusion mechanism leading to the formation of the gradient is reasonably well understood. Quantitative observations of this gradient in fixed tissue exhibit small fluctuations between neighboring nuclei, while the overall exponential profile ensures that such fluctuations can be monitored over a wide range of concentrations which extend to the lower limits of detectability.

The intrinsic fluctuations of the Bcd gradient will be well described by a stochastic Reaction-Diffusion Master Equation (RDME). Such equations typically do not have analytic solutions and are usually solved by running repeated stochastic simulations. A well known simulation algorithm due to Gillespie [13,14] performs an exact simulation of the Chemical Master Equation for a well mixed system. This method has been extended to spatially distributed systems by Elf and others [15,16]. These authors divide the spatially extended system into a series of subvolumes that are small enough to be regarded as well mixed. In each subvolume chemical reactions are simulated by Gillespie's algorithm, while diffusion between subvolumes is treated as a first order reaction.

In this study, we compare the results of such simulations to data in order to discover whether or not the data is sufficiently accurate as to exhibit the signature of a simple stochastic process. Stochastic processes underlying biological regulation can in general form complex patterns as a result of the reaction network, and for this reason a full consideration of 3 dimensional geometry is often necessary [15]. In the case considered here fluctuations occur passively in the course of diffusion and the statistical signature that we seek is independent of detailed geometry. For these reasons, we chose to model a 1 dimensional system.

## Results and Discussion

In the following, we first characterize the statistical properties of random variations in expression level between adjacent nuclei of individual embryos and compare them to the results of stochastic simulations. At the level of single embryos, we do not find a clear signature of stochastic processes in the data, but the need to separate spatially changing mean expression values from their variation limits the amount of statistical information that can be obtained from individual embryos. To address this problem, we consider ensembles of embryos, both over the whole dataset and over a restricted subset with low embryo-to-embryo variation.

In order to interpret these data, we introduce a stochastic model of the immunostaining procedure. We denote all random variables in this article with an upper hat but write a specific value without the hat.Thus, for example, n^j
 MathType@MTEF@5@5@+=feaafiart1ev1aaatCvAUfKttLearuWrP9MDH5MBPbIqV92AaeXatLxBI9gBaebbnrfifHhDYfgasaacPC6xNi=xH8viVGI8Gi=hEeeu0xXdbba9frFj0xb9qqpG0dXdb9aspeI8k8fiI+fsY=rqGqVepae9pg0db9vqaiVgFr0xfr=xfr=xc9adbaqaaeGacaGaaiaabeqaaeqabiWaaaGcbaGafmOBa4MbaKaadaWgaaWcbaGaemOAaOgabeaaaaa@2ED1@ denotes a random variable for the number of Bcd molecules in subvolume *j*, while *n*_*j *_denotes a particular value of this variable.

### Single embryo analysis in the logarithmic domain

**Figure 1:**
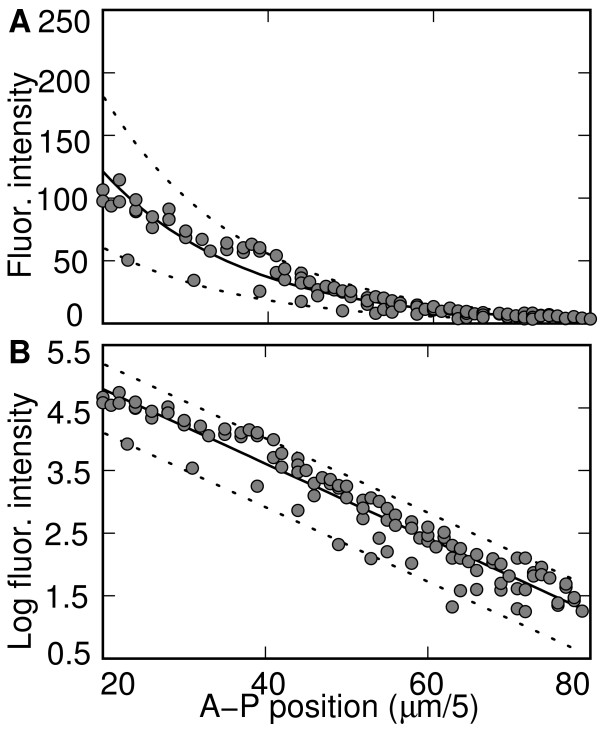
**Bicoid profile from FlyEx embryo ms18 after background removal**. (**A**) The spatial index *j *(*μ*m/5) is the index of 5 *μ*m bins. The fluorescence intensity *I*_*j *_(circles) were fit to an exponential F[*I*_*j*_] = *a *exp(-*j*/*λ*) (solid line) with two scaling index lines 1.5F[*I*_*j*_] and 0.5F[*I*_*j*_] (dashed). The fluorescence intensity were then converted into log scale in panel (**B**).

We find that the Bcd profiles of individual embryos observed by immunostaining (Fig. 1A) are exponential, as previously reported [17-19]. This profile strongly supports the model of an effective Fickian diffusion which gives an exponential decay solution at steady state [12,20]. Each embryo contains a collection of observations of expression (with variation) *I*_*j*_, and the observed properties of the exponential gradient will depend on the data through a function F embodying the least squares fitting procedure such that

F [*I*_*j*_] = *a *exp(-*j*/*λ*).

The residuals of the exponential vary from embryo to embryo in our data. In logarithmic coordinates the size of the residuals is independent of *j *for the anterior portion of the embryo, and in certain embryos (such as ms18) the size of the residuals is completely independent of *j *(Fig. 1B). For these embryos, the residuals are well described by

ln⁡(Ij)=ln⁡(a)−j/λ+W_,
 MathType@MTEF@5@5@+=feaafiart1ev1aaatCvAUfKttLearuWrP9MDH5MBPbIqV92AaeXatLxBI9gBaebbnrfifHhDYfgasaacPC6xNi=xI8qiVKYPFjYdHaVhbbf9v8qqaqFr0xc9vqFj0dXdbba91qpepeI8k8fiI+fsY=rqGqVepae9pg0db9vqaiVgFr0xfr=xfr=xc9adbaqaaeGacaGaaiaabeqaaeqabiWaaaGcbaGagiiBaWMaeiOBa4MaeiikaGIaemysaK0aaSbaaSqaaiabdQgaQbqabaGccqGGPaqkcqGH9aqpcyGGSbaBcqGGUbGBcqGGOaakcqWGHbqycqGGPaqkcqGHsislcqWGQbGAcqGGVaWliiGacqWF7oaBcqGHRaWkdaqiaaqaaiabdEfaxbGaayPadaGaeiilaWcaaa@42B6@

where W_
 MathType@MTEF@5@5@+=feaafiart1ev1aaatCvAUfKttLearuWrP9MDH5MBPbIqV92AaeXatLxBI9gBaebbnrfifHhDYfgasaacPC6xNi=xH8viVGI8Gi=hEeeu0xXdbba9frFj0xb9qqpG0dXdb9aspeI8k8fiI+fsY=rqGqVepae9pg0db9vqaiVgFr0xfr=xfr=xc9adbaqaaeGacaGaaiaabeqaaeqabiWaaaGcbaWaaecaaeaacqWGxbWvaiaawkWaaaaa@2DCC@ is an unknown random variable independent of *j*, so that the variance of ln(*I*_*j*_) is equal to the variance of W_
 MathType@MTEF@5@5@+=feaafiart1ev1aaatCvAUfKttLearuWrP9MDH5MBPbIqV92AaeXatLxBI9gBaebbnrfifHhDYfgasaacPC6xNi=xH8viVGI8Gi=hEeeu0xXdbba9frFj0xb9qqpG0dXdb9aspeI8k8fiI+fsY=rqGqVepae9pg0db9vqaiVgFr0xfr=xfr=xc9adbaqaaeGacaGaaiaabeqaaeqabiWaaaGcbaWaaecaaeaacqWGxbWvaiaawkWaaaaa@2DCC@.

### Intrinsic noise is insufficient

In order to understand whether the observed characteristic variation is a consequence of intrinsic fluctuations in Bcd molecular number, we performed stochastic simulations of an RDME which describes the time evolution of the Bcd gradient and compared them to data. We imagine the observed intensity *I*_*j *_to be a particular value of the random variable I^j
 MathType@MTEF@5@5@+=feaafiart1ev1aaatCvAUfKttLearuWrP9MDH5MBPbIqV92AaeXatLxBI9gBaebbnrfifHhDYfgasaacPC6xNi=xH8viVGI8Gi=hEeeu0xXdbba9frFj0xb9qqpG0dXdb9aspeI8k8fiI+fsY=rqGqVepae9pg0db9vqaiVgFr0xfr=xfr=xc9adbaqaaeGacaGaaiaabeqaaeqabiWaaaGcbaGafmysaKKbaKaadaWgaaWcbaGaemOAaOgabeaaaaa@2E87@. We further suppose that the random variable I^j
 MathType@MTEF@5@5@+=feaafiart1ev1aaatCvAUfKttLearuWrP9MDH5MBPbIqV92AaeXatLxBI9gBaebbnrfifHhDYfgasaacPC6xNi=xH8viVGI8Gi=hEeeu0xXdbba9frFj0xb9qqpG0dXdb9aspeI8k8fiI+fsY=rqGqVepae9pg0db9vqaiVgFr0xfr=xfr=xc9adbaqaaeGacaGaaiaabeqaaeqabiWaaaGcbaGafmysaKKbaKaadaWgaaWcbaGaemOAaOgabeaaaaa@2E87@ is determined by a direct linear rescaling of the Bcd molecular number such that

I^j=mn^j,
 MathType@MTEF@5@5@+=feaafiart1ev1aaatCvAUfKttLearuWrP9MDH5MBPbIqV92AaeXatLxBI9gBaebbnrfifHhDYfgasaacPC6xNi=xI8qiVKYPFjYdHaVhbbf9v8qqaqFr0xc9vqFj0dXdbba91qpepeI8k8fiI+fsY=rqGqVepae9pg0db9vqaiVgFr0xfr=xfr=xc9adbaqaaeGacaGaaiaabeqaaeqabiWaaaGcbaGafmysaKKbaKaadaWgaaWcbaGaemOAaOgabeaakiabg2da9iabd2gaTjqbd6gaUzaajaWaaSbaaSqaaiabdQgaQbqabaGccqGGSaalaaa@3530@

where the factor *m *represents the proportionality between one Bcd molecule and the corresponding fluorescence intensity, which includes the combined effects of tissue fixation, first and second antibody binding, fluorescence excitation and image processing.

As we do not know the exact *in vivo *system parameters and molecular number within each nucleus, we performed a complete inspection of the behavior of the variance of *m*n^j
 MathType@MTEF@5@5@+=feaafiart1ev1aaatCvAUfKttLearuWrP9MDH5MBPbIqV92AaeXatLxBI9gBaebbnrfifHhDYfgasaacPC6xNi=xH8viVGI8Gi=hEeeu0xXdbba9frFj0xb9qqpG0dXdb9aspeI8k8fiI+fsY=rqGqVepae9pg0db9vqaiVgFr0xfr=xfr=xc9adbaqaaeGacaGaaiaabeqaaeqabiWaaaGcbaGafmOBa4MbaKaadaWgaaWcbaGaemOAaOgabeaaaaa@2ED1@ in the four dimensional parameter space *f*(*m*, *J*, *D*, *ω*), where *J *is the synthesis rate of Bcd in the anterior compartment, *ω *is its decay rate, and *D *is the diffusion coefficient. It was always true that the residuals in logarithmic coordinate increased towards the posterior of the embryo, or in other words at lower levels of Bcd. This behavior strongly contrasts with the position-independent residuals seen in Figure 1 and described in equation (2).

### Measurement rescaling noise dominates

The simulation results suggest that intrinsic noise cannot explain the pattern of variance seen in Figure 1. Another possibility is the measurement process itself. In order to analyze this process, we consider a simple model of the measurement of fluorescence intensity, where

I^j=α^n^j+β^.
 MathType@MTEF@5@5@+=feaafiart1ev1aaatCvAUfKttLearuWrP9MDH5MBPbIqV92AaeXatLxBI9gBaebbnrfifHhDYfgasaacPC6xNi=xI8qiVKYPFjYdHaVhbbf9v8qqaqFr0xc9vqFj0dXdbba91qpepeI8k8fiI+fsY=rqGqVepae9pg0db9vqaiVgFr0xfr=xfr=xc9adbaqaaeGacaGaaiaabeqaaeqabiWaaaGcbaGafmysaKKbaKaadaWgaaWcbaGaemOAaOgabeaakiabg2da9GGaciqb=f7aHzaajaGafmOBa4MbaKaadaWgaaWcbaGaemOAaOgabeaakiabgUcaRiqb=j7aIzaajaacbaGae4Nla4caaa@381A@

Here α^
 MathType@MTEF@5@5@+=feaafiart1ev1aaatCvAUfKttLearuWrP9MDH5MBPbIqV92AaeXatLxBI9gBaebbnrfifHhDYfgasaacPC6xNi=xH8viVGI8Gi=hEeeu0xXdbba9frFj0xb9qqpG0dXdb9aspeI8k8fiI+fsY=rqGqVepae9pg0db9vqaiVgFr0xfr=xfr=xc9adbaqaaeGacaGaaiaabeqaaeqabiWaaaGcbaacciGaf8xSdeMbaKaaaaa@2D89@ is a spatially uniform random variable which replaces *m *in equation (3), and β^
 MathType@MTEF@5@5@+=feaafiart1ev1aaatCvAUfKttLearuWrP9MDH5MBPbIqV92AaeXatLxBI9gBaebbnrfifHhDYfgasaacPC6xNi=xH8viVGI8Gi=hEeeu0xXdbba9frFj0xb9qqpG0dXdb9aspeI8k8fiI+fsY=rqGqVepae9pg0db9vqaiVgFr0xfr=xfr=xc9adbaqaaeGacaGaaiaabeqaaeqabiWaaaGcbaacciGaf8NSdiMbaKaaaaa@2D8B@ is a spatially uniform random variable which represents nonspecific background staining. This picture allows us to consider noise that arises from both intrinsic and measurement related sources.

The simplest way to understand the consequences of (4) is to imagine the consequences if only one of α^
 MathType@MTEF@5@5@+=feaafiart1ev1aaatCvAUfKttLearuWrP9MDH5MBPbIqV92AaeXatLxBI9gBaebbnrfifHhDYfgasaacPC6xNi=xH8viVGI8Gi=hEeeu0xXdbba9frFj0xb9qqpG0dXdb9aspeI8k8fiI+fsY=rqGqVepae9pg0db9vqaiVgFr0xfr=xfr=xc9adbaqaaeGacaGaaiaabeqaaeqabiWaaaGcbaacciGaf8xSdeMbaKaaaaa@2D89@, n^j
 MathType@MTEF@5@5@+=feaafiart1ev1aaatCvAUfKttLearuWrP9MDH5MBPbIqV92AaeXatLxBI9gBaebbnrfifHhDYfgasaacPC6xNi=xH8viVGI8Gi=hEeeu0xXdbba9frFj0xb9qqpG0dXdb9aspeI8k8fiI+fsY=rqGqVepae9pg0db9vqaiVgFr0xfr=xfr=xc9adbaqaaeGacaGaaiaabeqaaeqabiWaaaGcbaGafmOBa4MbaKaadaWgaaWcbaGaemOAaOgabeaaaaa@2ED1@, and β^
 MathType@MTEF@5@5@+=feaafiart1ev1aaatCvAUfKttLearuWrP9MDH5MBPbIqV92AaeXatLxBI9gBaebbnrfifHhDYfgasaacPC6xNi=xH8viVGI8Gi=hEeeu0xXdbba9frFj0xb9qqpG0dXdb9aspeI8k8fiI+fsY=rqGqVepae9pg0db9vqaiVgFr0xfr=xfr=xc9adbaqaaeGacaGaaiaabeqaaeqabiWaaaGcbaacciGaf8NSdiMbaKaaaaa@2D8B@ are allowed to have finite variance while the other two are constrained to deterministic (zero variance) behavior. We have already pointed out that allowing finite variance for n^j
 MathType@MTEF@5@5@+=feaafiart1ev1aaatCvAUfKttLearuWrP9MDH5MBPbIqV92AaeXatLxBI9gBaebbnrfifHhDYfgasaacPC6xNi=xH8viVGI8Gi=hEeeu0xXdbba9frFj0xb9qqpG0dXdb9aspeI8k8fiI+fsY=rqGqVepae9pg0db9vqaiVgFr0xfr=xfr=xc9adbaqaaeGacaGaaiaabeqaaeqabiWaaaGcbaGafmOBa4MbaKaadaWgaaWcbaGaemOAaOgabeaaaaa@2ED1@ with α^
 MathType@MTEF@5@5@+=feaafiart1ev1aaatCvAUfKttLearuWrP9MDH5MBPbIqV92AaeXatLxBI9gBaebbnrfifHhDYfgasaacPC6xNi=xH8viVGI8Gi=hEeeu0xXdbba9frFj0xb9qqpG0dXdb9aspeI8k8fiI+fsY=rqGqVepae9pg0db9vqaiVgFr0xfr=xfr=xc9adbaqaaeGacaGaaiaabeqaaeqabiWaaaGcbaacciGaf8xSdeMbaKaaaaa@2D89@ and β^
 MathType@MTEF@5@5@+=feaafiart1ev1aaatCvAUfKttLearuWrP9MDH5MBPbIqV92AaeXatLxBI9gBaebbnrfifHhDYfgasaacPC6xNi=xH8viVGI8Gi=hEeeu0xXdbba9frFj0xb9qqpG0dXdb9aspeI8k8fiI+fsY=rqGqVepae9pg0db9vqaiVgFr0xfr=xfr=xc9adbaqaaeGacaGaaiaabeqaaeqabiWaaaGcbaacciGaf8NSdiMbaKaaaaa@2D8B@ deterministic leads to an increase of variance towards the posterior. The same is true if all noise comes from β^
 MathType@MTEF@5@5@+=feaafiart1ev1aaatCvAUfKttLearuWrP9MDH5MBPbIqV92AaeXatLxBI9gBaebbnrfifHhDYfgasaacPC6xNi=xH8viVGI8Gi=hEeeu0xXdbba9frFj0xb9qqpG0dXdb9aspeI8k8fiI+fsY=rqGqVepae9pg0db9vqaiVgFr0xfr=xfr=xc9adbaqaaeGacaGaaiaabeqaaeqabiWaaaGcbaacciGaf8NSdiMbaKaaaaa@2D8B@, that is if noise from background staining dominates. This will also lead to more noise in the logarithmic domain towards the posterior as β^
 MathType@MTEF@5@5@+=feaafiart1ev1aaatCvAUfKttLearuWrP9MDH5MBPbIqV92AaeXatLxBI9gBaebbnrfifHhDYfgasaacPC6xNi=xH8viVGI8Gi=hEeeu0xXdbba9frFj0xb9qqpG0dXdb9aspeI8k8fiI+fsY=rqGqVepae9pg0db9vqaiVgFr0xfr=xfr=xc9adbaqaaeGacaGaaiaabeqaaeqabiWaaaGcbaacciGaf8NSdiMbaKaaaaa@2D8B@ provides a larger proportion of the total detected signal. Let us next consider the case where all noise comes from α^
 MathType@MTEF@5@5@+=feaafiart1ev1aaatCvAUfKttLearuWrP9MDH5MBPbIqV92AaeXatLxBI9gBaebbnrfifHhDYfgasaacPC6xNi=xH8viVGI8Gi=hEeeu0xXdbba9frFj0xb9qqpG0dXdb9aspeI8k8fiI+fsY=rqGqVepae9pg0db9vqaiVgFr0xfr=xfr=xc9adbaqaaeGacaGaaiaabeqaaeqabiWaaaGcbaacciGaf8xSdeMbaKaaaaa@2D89@.

In order to understand the role of α^
 MathType@MTEF@5@5@+=feaafiart1ev1aaatCvAUfKttLearuWrP9MDH5MBPbIqV92AaeXatLxBI9gBaebbnrfifHhDYfgasaacPC6xNi=xH8viVGI8Gi=hEeeu0xXdbba9frFj0xb9qqpG0dXdb9aspeI8k8fiI+fsY=rqGqVepae9pg0db9vqaiVgFr0xfr=xfr=xc9adbaqaaeGacaGaaiaabeqaaeqabiWaaaGcbaacciGaf8xSdeMbaKaaaaa@2D89@, note that the spatial pattern of variance observed in Figure 1 can be captured by an exponential function multiplied by a normal random variable. This suggests that the simplest picture for (4) is given by assuming that there is no background noise β^
 MathType@MTEF@5@5@+=feaafiart1ev1aaatCvAUfKttLearuWrP9MDH5MBPbIqV92AaeXatLxBI9gBaebbnrfifHhDYfgasaacPC6xNi=xH8viVGI8Gi=hEeeu0xXdbba9frFj0xb9qqpG0dXdb9aspeI8k8fiI+fsY=rqGqVepae9pg0db9vqaiVgFr0xfr=xfr=xc9adbaqaaeGacaGaaiaabeqaaeqabiWaaaGcbaacciGaf8NSdiMbaKaaaaa@2D8B@ and no intrinsic noise for Bcd so that n^j=〈n^j〉
 MathType@MTEF@5@5@+=feaafiart1ev1aaatCvAUfKttLearuWrP9MDH5MBPbIqV92AaeXatLxBI9gBaebbnrfifHhDYfgasaacPC6xNi=xH8viVGI8Gi=hEeeu0xXdbba9frFj0xb9qqpG0dXdb9aspeI8k8fiI+fsY=rqGqVepae9pg0db9vqaiVgFr0xfr=xfr=xc9adbaqaaeGacaGaaiaabeqaaeqabiWaaaGcbaGafmOBa4MbaKaadaWgaaWcbaGaemOAaOgabeaakiabg2da9iabgMYiHlqbd6gaUzaajaWaaSbaaSqaaiabdQgaQbqabaGccqGHQms8aaa@366C@. Moreover, it suggests that the rescaling noise α^
 MathType@MTEF@5@5@+=feaafiart1ev1aaatCvAUfKttLearuWrP9MDH5MBPbIqV92AaeXatLxBI9gBaebbnrfifHhDYfgasaacPC6xNi=xH8viVGI8Gi=hEeeu0xXdbba9frFj0xb9qqpG0dXdb9aspeI8k8fiI+fsY=rqGqVepae9pg0db9vqaiVgFr0xfr=xfr=xc9adbaqaaeGacaGaaiaabeqaaeqabiWaaaGcbaacciGaf8xSdeMbaKaaaaa@2D89@ from measurement uncertainty is uniform across the embryo (independent of *j*) and is normally distributed with

α^=m(1+σαN^(0,1)),
 MathType@MTEF@5@5@+=feaafiart1ev1aaatCvAUfKttLearuWrP9MDH5MBPbIqV92AaeXatLxBI9gBaebbnrfifHhDYfgasaacPC6xNi=xI8qiVKYPFjYdHaVhbbf9v8qqaqFr0xc9vqFj0dXdbba91qpepeI8k8fiI+fsY=rqGqVepae9pg0db9vqaiVgFr0xfr=xfr=xc9adbaqaaeGacaGaaiaabeqaaeqabiWaaaGcbaacciGaf8xSdeMbaKaacqGH9aqpcqWGTbqBcqGGOaakcqaIXaqmcqGHRaWkcqWFdpWCdaWgaaWcbaGae8xSdegabeaakiqbd6eaozaajaGaeiikaGIaeGimaaJaeiilaWIaeGymaeJaeiykaKIaeiykaKIaeiilaWcaaa@3DD7@

where N^(0,1)
 MathType@MTEF@5@5@+=feaafiart1ev1aaatCvAUfKttLearuWrP9MDH5MBPbIqV92AaeXatLxBI9gBaebbnrfifHhDYfgasaacPC6xNi=xH8viVGI8Gi=hEeeu0xXdbba9frFj0xb9qqpG0dXdb9aspeI8k8fiI+fsY=rqGqVepae9pg0db9vqaiVgFr0xfr=xfr=xc9adbaqaaeGacaGaaiaabeqaaeqabiWaaaGcbaGafmOta4KbaKaacqGGOaakcqaIWaamcqGGSaalcqaIXaqmcqGGPaqkaaa@3178@ is a normal independent random variable with mean 0 and variance 1. Then we can model I^j
 MathType@MTEF@5@5@+=feaafiart1ev1aaatCvAUfKttLearuWrP9MDH5MBPbIqV92AaeXatLxBI9gBaebbnrfifHhDYfgasaacPC6xNi=xH8viVGI8Gi=hEeeu0xXdbba9frFj0xb9qqpG0dXdb9aspeI8k8fiI+fsY=rqGqVepae9pg0db9vqaiVgFr0xfr=xfr=xc9adbaqaaeGacaGaaiaabeqaaeqabiWaaaGcbaGafmysaKKbaKaadaWgaaWcbaGaemOAaOgabeaaaaa@2E87@ by

I^j=α^〈n^j〉.
 MathType@MTEF@5@5@+=feaafiart1ev1aaatCvAUfKttLearuWrP9MDH5MBPbIqV92AaeXatLxBI9gBaebbnrfifHhDYfgasaacPC6xNi=xI8qiVKYPFjYdHaVhbbf9v8qqaqFr0xc9vqFj0dXdbba91qpepeI8k8fiI+fsY=rqGqVepae9pg0db9vqaiVgFr0xfr=xfr=xc9adbaqaaeGacaGaaiaabeqaaeqabiWaaaGcbaGafmysaKKbaKaadaWgaaWcbaGaemOAaOgabeaakiabg2da9GGaciqb=f7aHzaajaGaeyykJeUafmOBa4MbaKaadaWgaaWcbaGaemOAaOgabeaakiabgQYiXlabc6caUaaa@390A@

In steady state, 〈n^j〉
 MathType@MTEF@5@5@+=feaafiart1ev1aaatCvAUfKttLearuWrP9MDH5MBPbIqV92AaeXatLxBI9gBaebbnrfifHhDYfgasaacPC6xNi=xH8viVGI8Gi=hEeeu0xXdbba9frFj0xb9qqpG0dXdb9aspeI8k8fiI+fsY=rqGqVepae9pg0db9vqaiVgFr0xfr=xfr=xc9adbaqaaeGacaGaaiaabeqaaeqabiWaaaGcbaGaeyykJeUafmOBa4MbaKaadaWgaaWcbaGaemOAaOgabeaakiabgQYiXdaa@325E@ = *a*/*m *exp(-*j*/*λ*). Taking logarithms allows us to write

ln⁡(I^j)=ln⁡(a)−j/λ+ln⁡(1+σαN^(0,1)).
 MathType@MTEF@5@5@+=feaafiart1ev1aaatCvAUfKttLearuWrP9MDH5MBPbIqV92AaeXatLxBI9gBaebbnrfifHhDYfgasaacPC6xNi=xI8qiVKYPFjYdHaVhbbf9v8qqaqFr0xc9vqFj0dXdbba91qpepeI8k8fiI+fsY=rqGqVepae9pg0db9vqaiVgFr0xfr=xfr=xc9adbaqaaeGacaGaaiaabeqaaeqabiWaaaGcbaGagiiBaWMaeiOBa4MaeiikaGIafmysaKKbaKaadaWgaaWcbaGaemOAaOgabeaakiabcMcaPiabg2da9iGbcYgaSjabc6gaUjabcIcaOiabdggaHjabcMcaPiabgkHiTiabdQgaQjabc+caVGGaciab=T7aSjabgUcaRiGbcYgaSjabc6gaUjabcIcaOiabigdaXiabgUcaRiab=n8aZnaaBaaaleaacqWFXoqyaeqaaOGafmOta4KbaKaacqGGOaakcqaIWaamcqGGSaalcqaIXaqmcqGGPaqkcqGGPaqkcqGGUaGlaaa@504E@

Comparison with equation (2) indicates that W_
 MathType@MTEF@5@5@+=feaafiart1ev1aaatCvAUfKttLearuWrP9MDH5MBPbIqV92AaeXatLxBI9gBaebbnrfifHhDYfgasaacPC6xNi=xH8viVGI8Gi=hEeeu0xXdbba9frFj0xb9qqpG0dXdb9aspeI8k8fiI+fsY=rqGqVepae9pg0db9vqaiVgFr0xfr=xfr=xc9adbaqaaeGacaGaaiaabeqaaeqabiWaaaGcbaWaaecaaeaacqWGxbWvaiaawkWaaaaa@2DCC@ = ln(1 + *σ*_*α *_N^(0,1)
 MathType@MTEF@5@5@+=feaafiart1ev1aaatCvAUfKttLearuWrP9MDH5MBPbIqV92AaeXatLxBI9gBaebbnrfifHhDYfgasaacPC6xNi=xH8viVGI8Gi=hEeeu0xXdbba9frFj0xb9qqpG0dXdb9aspeI8k8fiI+fsY=rqGqVepae9pg0db9vqaiVgFr0xfr=xfr=xc9adbaqaaeGacaGaaiaabeqaaeqabiWaaaGcbaGafmOta4KbaKaacqGGOaakcqaIWaamcqGGSaalcqaIXaqmcqGGPaqkaaa@3178@).

The reason we do not see intrinsic noise in this individual embryo is most likely low measurement sensitivity, that is to say a low molecule-to-fluorescence mean rescaling ratio *m*. When *m *is small enough, it can mask the variance of n^j
 MathType@MTEF@5@5@+=feaafiart1ev1aaatCvAUfKttLearuWrP9MDH5MBPbIqV92AaeXatLxBI9gBaebbnrfifHhDYfgasaacPC6xNi=xH8viVGI8Gi=hEeeu0xXdbba9frFj0xb9qqpG0dXdb9aspeI8k8fiI+fsY=rqGqVepae9pg0db9vqaiVgFr0xfr=xfr=xc9adbaqaaeGacaGaaiaabeqaaeqabiWaaaGcbaGafmOBa4MbaKaadaWgaaWcbaGaemOAaOgabeaaaaa@2ED1@ because the variance of the rescaled gradient is given by

var⁡(mn^j)=m2var⁡(n^j).
 MathType@MTEF@5@5@+=feaafiart1ev1aaatCvAUfKttLearuWrP9MDH5MBPbIqV92AaeXatLxBI9gBaebbnrfifHhDYfgasaacPC6xNi=xI8qiVKYPFjYdHaVhbbf9v8qqaqFr0xc9vqFj0dXdbba91qpepeI8k8fiI+fsY=rqGqVepae9pg0db9vqaiVgFr0xfr=xfr=xc9adbaqaaeGacaGaaiaabeqaaeqabiWaaaGcbaGagiODayNaeiyyaeMaeiOCaiNaeiikaGIaemyBa0MafmOBa4MbaKaadaWgaaWcbaGaemOAaOgabeaakiabcMcaPiabg2da9iabd2gaTnaaCaaaleqabaGaeGOmaidaaOGagiODayNaeiyyaeMaeiOCaiNaeiikaGIafmOBa4MbaKaadaWgaaWcbaGaemOAaOgabeaakiabcMcaPiabc6caUaaa@43C6@

As *m*^2 ^→ 0 with var(n^j
 MathType@MTEF@5@5@+=feaafiart1ev1aaatCvAUfKttLearuWrP9MDH5MBPbIqV92AaeXatLxBI9gBaebbnrfifHhDYfgasaacPC6xNi=xH8viVGI8Gi=hEeeu0xXdbba9frFj0xb9qqpG0dXdb9aspeI8k8fiI+fsY=rqGqVepae9pg0db9vqaiVgFr0xfr=xfr=xc9adbaqaaeGacaGaaiaabeqaaeqabiWaaaGcbaGafmOBa4MbaKaadaWgaaWcbaGaemOAaOgabeaaaaa@2ED1@) bounded in a reasonable way throughout the embryo, we will get var(*m*n^j
 MathType@MTEF@5@5@+=feaafiart1ev1aaatCvAUfKttLearuWrP9MDH5MBPbIqV92AaeXatLxBI9gBaebbnrfifHhDYfgasaacPC6xNi=xH8viVGI8Gi=hEeeu0xXdbba9frFj0xb9qqpG0dXdb9aspeI8k8fiI+fsY=rqGqVepae9pg0db9vqaiVgFr0xfr=xfr=xc9adbaqaaeGacaGaaiaabeqaaeqabiWaaaGcbaGafmOBa4MbaKaadaWgaaWcbaGaemOAaOgabeaaaaa@2ED1@) → 0. Hence the rescaled gradient *m*n^j
 MathType@MTEF@5@5@+=feaafiart1ev1aaatCvAUfKttLearuWrP9MDH5MBPbIqV92AaeXatLxBI9gBaebbnrfifHhDYfgasaacPC6xNi=xH8viVGI8Gi=hEeeu0xXdbba9frFj0xb9qqpG0dXdb9aspeI8k8fiI+fsY=rqGqVepae9pg0db9vqaiVgFr0xfr=xfr=xc9adbaqaaeGacaGaaiaabeqaaeqabiWaaaGcbaGafmOBa4MbaKaadaWgaaWcbaGaemOAaOgabeaaaaa@2ED1@ can be treated as deterministic by letting

mn^j=〈mn^j〉=m〈n^j〉.
 MathType@MTEF@5@5@+=feaafiart1ev1aaatCvAUfKttLearuWrP9MDH5MBPbIqV92AaeXatLxBI9gBaebbnrfifHhDYfgasaacPC6xNi=xI8qiVKYPFjYdHaVhbbf9v8qqaqFr0xc9vqFj0dXdbba91qpepeI8k8fiI+fsY=rqGqVepae9pg0db9vqaiVgFr0xfr=xfr=xc9adbaqaaeGacaGaaiaabeqaaeqabiWaaaGcbaGaemyBa0MafmOBa4MbaKaadaWgaaWcbaGaemOAaOgabeaakiabg2da9iabgMYiHlabd2gaTjqbd6gaUzaajaWaaSbaaSqaaiabdQgaQbqabaGccqGHQms8cqGH9aqpcqWGTbqBcqGHPms4cuWGUbGBgaqcamaaBaaaleaacqWGQbGAaeqaaOGaeyOkJeVaeiOla4caaa@4358@

In summary, we suspect the nucleus-to-nucleus variation observed in our data comes chiefly from the experimental rescaling noise α^
 MathType@MTEF@5@5@+=feaafiart1ev1aaatCvAUfKttLearuWrP9MDH5MBPbIqV92AaeXatLxBI9gBaebbnrfifHhDYfgasaacPC6xNi=xH8viVGI8Gi=hEeeu0xXdbba9frFj0xb9qqpG0dXdb9aspeI8k8fiI+fsY=rqGqVepae9pg0db9vqaiVgFr0xfr=xfr=xc9adbaqaaeGacaGaaiaabeqaaeqabiWaaaGcbaacciGaf8xSdeMbaKaaaaa@2D89@, which is normally distributed. If Bcd intrinsic noise is to be observed, then the fluorescence noise intensity should be a function of the mean intensity in logarithm, instead of a constant as observed in embryo ms18. Nevertheless, the necessity of considering data in spatially resolved bins limits the amount of information that can be obtained from a single embryo. More information can be obtained by pooling data from many embryos, and we discuss this point in the next section.

### Statistical analysis of an ensemble of embryos

Statistical analysis of many embryos is required in order to take our analysis further. This analysis will show how physical constraints on the model can be inferred from the ensemble dataset, and independent random variables separated. We consider a set of embryos indexed by *i *with expression levels *I*_*ij*_, and then pool data from corresponding bins to obtain the ensemble dataset ∪iIij
 MathType@MTEF@5@5@+=feaafiart1ev1aaatCvAUfKttLearuWrP9MDH5MBPbIqV92AaeXatLxBI9gBaebbnrfifHhDYfgasaacPC6xNi=xH8viVGI8Gi=hEeeu0xXdbba9frFj0xb9qqpG0dXdb9aspeI8k8fiI+fsY=rqGqVepae9pg0db9vqaiVgFr0xfr=xfr=xc9adbaqaaeGacaGaaiaabeqaaeqabiWaaaGcbaWaambuaeaacqWGjbqsdaWgaaWcbaGaemyAaKMaemOAaOgabeaaaeaacqWGPbqAaeqaniablQIivbaaaa@32D6@. Since this dataset includes embryo-to-embryo variability, i.e. the variation of system parameters and experimental conditions over different embryos, the variance of the ensemble profile will be an upper bound for the average variance within each embryo. This allows us to identify physical constraints for system parameters and to determine if the model behaves properly within the permitted range of parameters.

We define independent global random variables I^j=α^n^j+β^
 MathType@MTEF@5@5@+=feaafiart1ev1aaatCvAUfKttLearuWrP9MDH5MBPbIqV92AaeXatLxBI9gBaebbnrfifHhDYfgasaacPC6xNi=xH8viVGI8Gi=hEeeu0xXdbba9frFj0xb9qqpG0dXdb9aspeI8k8fiI+fsY=rqGqVepae9pg0db9vqaiVgFr0xfr=xfr=xc9adbaqaaeGacaGaaiaabeqaaeqabiWaaaGcbaGafmysaKKbaKaadaWgaaWcbaGaemOAaOgabeaakiabg2da9GGaciqb=f7aHzaajaGafmOBa4MbaKaadaWgaaWcbaGaemOAaOgabeaakiabgUcaRiqb=j7aIzaajaaaaa@36E3@ as described in the last section with normal distributed measurement uncertainty α^
 MathType@MTEF@5@5@+=feaafiart1ev1aaatCvAUfKttLearuWrP9MDH5MBPbIqV92AaeXatLxBI9gBaebbnrfifHhDYfgasaacPC6xNi=xH8viVGI8Gi=hEeeu0xXdbba9frFj0xb9qqpG0dXdb9aspeI8k8fiI+fsY=rqGqVepae9pg0db9vqaiVgFr0xfr=xfr=xc9adbaqaaeGacaGaaiaabeqaaeqabiWaaaGcbaacciGaf8xSdeMbaKaaaaa@2D89@ = *m *(1 + *σ*_*α *_N^(0,1)
 MathType@MTEF@5@5@+=feaafiart1ev1aaatCvAUfKttLearuWrP9MDH5MBPbIqV92AaeXatLxBI9gBaebbnrfifHhDYfgasaacPC6xNi=xH8viVGI8Gi=hEeeu0xXdbba9frFj0xb9qqpG0dXdb9aspeI8k8fiI+fsY=rqGqVepae9pg0db9vqaiVgFr0xfr=xfr=xc9adbaqaaeGacaGaaiaabeqaaeqabiWaaaGcbaGafmOta4KbaKaacqGGOaakcqaIWaamcqGGSaalcqaIXaqmcqGGPaqkaaa@3178@). We also now assume that background noise is normally distributed with β^
 MathType@MTEF@5@5@+=feaafiart1ev1aaatCvAUfKttLearuWrP9MDH5MBPbIqV92AaeXatLxBI9gBaebbnrfifHhDYfgasaacPC6xNi=xH8viVGI8Gi=hEeeu0xXdbba9frFj0xb9qqpG0dXdb9aspeI8k8fiI+fsY=rqGqVepae9pg0db9vqaiVgFr0xfr=xfr=xc9adbaqaaeGacaGaaiaabeqaaeqabiWaaaGcbaacciGaf8NSdiMbaKaaaaa@2D8B@ = *σ*_*β *_N^(0,1)
 MathType@MTEF@5@5@+=feaafiart1ev1aaatCvAUfKttLearuWrP9MDH5MBPbIqV92AaeXatLxBI9gBaebbnrfifHhDYfgasaacPC6xNi=xH8viVGI8Gi=hEeeu0xXdbba9frFj0xb9qqpG0dXdb9aspeI8k8fiI+fsY=rqGqVepae9pg0db9vqaiVgFr0xfr=xfr=xc9adbaqaaeGacaGaaiaabeqaaeqabiWaaaGcbaGafmOta4KbaKaacqGGOaakcqaIWaamcqGGSaalcqaIXaqmcqGGPaqkaaa@3178@. We assume such global random variable represent the average variability for each embryo. The statistics of simulated global random variables were then collected from 2000 stochastic simulation runs and the Bcd molecular number random variable n^j
 MathType@MTEF@5@5@+=feaafiart1ev1aaatCvAUfKttLearuWrP9MDH5MBPbIqV92AaeXatLxBI9gBaebbnrfifHhDYfgasaacPC6xNi=xH8viVGI8Gi=hEeeu0xXdbba9frFj0xb9qqpG0dXdb9aspeI8k8fiI+fsY=rqGqVepae9pg0db9vqaiVgFr0xfr=xfr=xc9adbaqaaeGacaGaaiaabeqaaeqabiWaaaGcbaGafmOBa4MbaKaadaWgaaWcbaGaemOAaOgabeaaaaa@2ED1@ were sampled after reaching steady state. In this section we explicitly consider the effects of different choices for the molecule-to-fluorescence rescaling ratio *m *by comparing simulations to data at differing values of this parameter. Because *m *is not an explicit input to the model, this comparison is effected by varying the synthesis rate *J*, which varies the molecular number, and comparing the behavior of the model to fluorescence data which is on a fixed but arbitrary scale.

We seek optimal values of *m *such that the simulated global random variable I^j
 MathType@MTEF@5@5@+=feaafiart1ev1aaatCvAUfKttLearuWrP9MDH5MBPbIqV92AaeXatLxBI9gBaebbnrfifHhDYfgasaacPC6xNi=xH8viVGI8Gi=hEeeu0xXdbba9frFj0xb9qqpG0dXdb9aspeI8k8fiI+fsY=rqGqVepae9pg0db9vqaiVgFr0xfr=xfr=xc9adbaqaaeGacaGaaiaabeqaaeqabiWaaaGcbaGafmysaKKbaKaadaWgaaWcbaGaemOAaOgabeaaaaa@2E87@ is constrained by the variation observed in the immunostained ensemble data ∪iIij
 MathType@MTEF@5@5@+=feaafiart1ev1aaatCvAUfKttLearuWrP9MDH5MBPbIqV92AaeXatLxBI9gBaebbnrfifHhDYfgasaacPC6xNi=xH8viVGI8Gi=hEeeu0xXdbba9frFj0xb9qqpG0dXdb9aspeI8k8fiI+fsY=rqGqVepae9pg0db9vqaiVgFr0xfr=xfr=xc9adbaqaaeGacaGaaiaabeqaaeqabiWaaaGcbaWaambuaeaacqWGjbqsdaWgaaWcbaGaemyAaKMaemOAaOgabeaaaeaacqWGPbqAaeqaniablQIivbaaaa@32D6@ by the condition

〈I^j〉=〈∪iIij〉
 MathType@MTEF@5@5@+=feaafiart1ev1aaatCvAUfKttLearuWrP9MDH5MBPbIqV92AaeXatLxBI9gBaebbnrfifHhDYfgasaacPC6xNi=xI8qiVKYPFjYdHaVhbbf9v8qqaqFr0xc9vqFj0dXdbba91qpepeI8k8fiI+fsY=rqGqVepae9pg0db9vqaiVgFr0xfr=xfr=xc9adbaqaaeGacaGaaiaabeqaaeqabiWaaaGcbaGaeyykJeUafmysaKKbaKaadaWgaaWcbaGaemOAaOgabeaakiabgQYiXlabg2da9iabgMYiHpaatafabaGaemysaK0aaSbaaSqaaiabdMgaPjabdQgaQbqabaaabaGaemyAaKgabeqdcqWIQisvaOGaeyOkJepaaa@3DF8@

var⁡(I^j)≤var⁡(∪iIij).
 MathType@MTEF@5@5@+=feaafiart1ev1aaatCvAUfKttLearuWrP9MDH5MBPbIqV92AaeXatLxBI9gBaebbnrfifHhDYfgasaacPC6xNi=xI8qiVKYPFjYdHaVhbbf9v8qqaqFr0xc9vqFj0dXdbba91qpepeI8k8fiI+fsY=rqGqVepae9pg0db9vqaiVgFr0xfr=xfr=xc9adbaqaaeGacaGaaiaabeqaaeqabiWaaaGcbaGagiODayNaeiyyaeMaeiOCaiNaeiikaGIafmysaKKbaKaadaWgaaWcbaGaemOAaOgabeaakiabcMcaPiabgsMiJkGbcAha2jabcggaHjabckhaYjabcIcaOmaatafabaGaemysaK0aaSbaaSqaaiabdMgaPjabdQgaQbqabaaabaGaemyAaKgabeqdcqWIQisvaOGaeiykaKIaeiOla4caaa@4441@

Because comparison with mean and variance of the ensemble data is not straightforward, we approximate the above conditions by comparing their exponential fit, F, and the normalized variance *η*^2^, where *η*^2 ^= var(*I*)/⟨*I*⟩^2^, and *I *= I^j
 MathType@MTEF@5@5@+=feaafiart1ev1aaatCvAUfKttLearuWrP9MDH5MBPbIqV92AaeXatLxBI9gBaebbnrfifHhDYfgasaacPC6xNi=xH8viVGI8Gi=hEeeu0xXdbba9frFj0xb9qqpG0dXdb9aspeI8k8fiI+fsY=rqGqVepae9pg0db9vqaiVgFr0xfr=xfr=xc9adbaqaaeGacaGaaiaabeqaaeqabiWaaaGcbaGafmysaKKbaKaadaWgaaWcbaGaemOAaOgabeaaaaa@2E87@ or *I*_*ij *_for simulation and data respectively. Then (7a) and (7b) become

〈I^j〉=F[∪iIij]
 MathType@MTEF@5@5@+=feaafiart1ev1aaatCvAUfKttLearuWrP9MDH5MBPbIqV92AaeXatLxBI9gBaebbnrfifHhDYfgasaacPC6xNi=xI8qiVKYPFjYdHaVhbbf9v8qqaqFr0xc9vqFj0dXdbba91qpepeI8k8fiI+fsY=rqGqVepae9pg0db9vqaiVgFr0xfr=xfr=xc9adbaqaaeGacaGaaiaabeqaaeqabiWaaaGcbaGaeyykJeUafmysaKKbaKaadaWgaaWcbaGaemOAaOgabeaakiabgQYiXlabg2da9iabbAeagjabcUfaBnaatafabaGaemysaK0aaSbaaSqaaiabdMgaPjabdQgaQbqabaaabaGaemyAaKgabeqdcqWIQisvaOGaeiyxa0faaa@3E08@

η2(I^j)≤η2(∪iIij).
 MathType@MTEF@5@5@+=feaafiart1ev1aaatCvAUfKttLearuWrP9MDH5MBPbIqV92AaeXatLxBI9gBaebbnrfifHhDYfgasaacPC6xNi=xI8qiVKYPFjYdHaVhbbf9v8qqaqFr0xc9vqFj0dXdbba91qpepeI8k8fiI+fsY=rqGqVepae9pg0db9vqaiVgFr0xfr=xfr=xc9adbaqaaeGacaGaaiaabeqaaeqabiWaaaGcbaacciGae83TdG2aaWbaaSqabeaacqaIYaGmaaGccqGGOaakcuWGjbqsgaqcamaaBaaaleaacqWGQbGAaeqaaOGaeiykaKIaeyizImQae83TdG2aaWbaaSqabeaacqaIYaGmaaGccqGGOaakdaWeqbqaaiabdMeajnaaBaaaleaacqWGPbqAcqWGQbGAaeqaaaqaaiabdMgaPbqab0GaeSOkIufakiabcMcaPiabc6caUaaa@4195@

In (8a), ⟨I^j
 MathType@MTEF@5@5@+=feaafiart1ev1aaatCvAUfKttLearuWrP9MDH5MBPbIqV92AaeXatLxBI9gBaebbnrfifHhDYfgasaacPC6xNi=xH8viVGI8Gi=hEeeu0xXdbba9frFj0xb9qqpG0dXdb9aspeI8k8fiI+fsY=rqGqVepae9pg0db9vqaiVgFr0xfr=xfr=xc9adbaqaaeGacaGaaiaabeqaaeqabiWaaaGcbaGafmysaKKbaKaadaWgaaWcbaGaemOAaOgabeaaaaa@2E87@⟩ = *ma' *exp(-*j*/*λ'*) from simulation and F[∪iIij]=aexp⁡(−j/λ)
 MathType@MTEF@5@5@+=feaafiart1ev1aaatCvAUfKttLearuWrP9MDH5MBPbIqV92AaeXatLxBI9gBaebbnrfifHhDYfgasaacPC6xNi=xI8qiVKYPFjYdHaVhbbf9v8qqaqFr0xc9vqFj0dXdbba91qpepeI8k8fiI+fsY=rqGqVepae9pg0db9vqaiVgFr0xfr=xfr=xc9adbaqaaeGacaGaaiaabeqaaeqabiWaaaGcbaGaeeOrayKaei4waS1aambuaeaacqWGjbqsdaWgaaWcbaGaemyAaKMaemOAaOgabeaaaeaacqWGPbqAaeqaniablQIivbGccqGGDbqxcqGH9aqpcqWGHbqycyGGLbqzcqGG4baEcqGGWbaCcqGGOaakcqGHsislcqWGQbGAcqGGVaWliiGacqWF7oaBcqGGPaqkaaa@43E3@ from the ensemble data. Because *λ' *is only determined by *D *and *ω*, we first select combinations of *D *and *ω *such that *λ' *= *λ*. Selecting a synthesis rate *J *determines *a*', and also *m*, because *m *= *a/a'*. Finally, biologically reasonable values of *J *are determined by the constraint (8b).

We seek to establish which terms of equation (4) dominate the observed variance in different parts of the embryo. To do this, we will graphically compare the total observed variance with a set of simulations in which variance arises from different subsets of the random variables in (4). We denote these restricted models by placing brackets around the subset of random variables which contribute to the variance. Thus the full model in (4) can be denoted by I^j=[αnβ_]
 MathType@MTEF@5@5@+=feaafiart1ev1aaatCvAUfKttLearuWrP9MDH5MBPbIqV92AaeXatLxBI9gBaebbnrfifHhDYfgasaacPC6xNi=xH8viVGI8Gi=hEeeu0xXdbba9frFj0xb9qqpG0dXdb9aspeI8k8fiI+fsY=rqGqVepae9pg0db9vqaiVgFr0xfr=xfr=xc9adbaqaaeGacaGaaiaabeqaaeqabiWaaaGcbaGafmysaKKbaKaadaWgaaWcbaGaemOAaOgabeaakiabg2da9iabcUfaBnaaHaaabaacciGae8xSdeMaemOBa4Mae8NSdigacaGLcmaacqGGDbqxaaa@3780@. A model with no variance contributed by background is denoted by [αn_]=α^n^j
 MathType@MTEF@5@5@+=feaafiart1ev1aaatCvAUfKttLearuWrP9MDH5MBPbIqV92AaeXatLxBI9gBaebbnrfifHhDYfgasaacPC6xNi=xH8viVGI8Gi=hEeeu0xXdbba9frFj0xb9qqpG0dXdb9aspeI8k8fiI+fsY=rqGqVepae9pg0db9vqaiVgFr0xfr=xfr=xc9adbaqaaeGacaGaaiaabeqaaeqabiWaaaGcbaGaei4waS1aaecaaeaaiiGacqWFXoqycqWGUbGBaiaawkWaaiabc2faDjabg2da9iqb=f7aHzaajaGafmOBa4MbaKaadaWgaaWcbaGaemOAaOgabeaaaaa@37CE@, while models in which all variance is contributed only by rescaling, background, or molecular number are denoted respectively by [α^]=α^〈n^j〉
 MathType@MTEF@5@5@+=feaafiart1ev1aaatCvAUfKttLearuWrP9MDH5MBPbIqV92AaeXatLxBI9gBaebbnrfifHhDYfgasaacPC6xNi=xH8viVGI8Gi=hEeeu0xXdbba9frFj0xb9qqpG0dXdb9aspeI8k8fiI+fsY=rqGqVepae9pg0db9vqaiVgFr0xfr=xfr=xc9adbaqaaeGacaGaaiaabeqaaeqabiWaaaGcbaGaei4waSfcciGaf8xSdeMbaKaacqGGDbqxcqGH9aqpcuWFXoqygaqcaiabgMYiHlqbd6gaUzaajaWaaSbaaSqaaiabdQgaQbqabaGccqGHQms8aaa@3944@, [β^]=m〈n^j〉+β^
 MathType@MTEF@5@5@+=feaafiart1ev1aaatCvAUfKttLearuWrP9MDH5MBPbIqV92AaeXatLxBI9gBaebbnrfifHhDYfgasaacPC6xNi=xH8viVGI8Gi=hEeeu0xXdbba9frFj0xb9qqpG0dXdb9aspeI8k8fiI+fsY=rqGqVepae9pg0db9vqaiVgFr0xfr=xfr=xc9adbaqaaeGacaGaaiaabeqaaeqabiWaaaGcbaGaei4waSfcciGaf8NSdiMbaKaacqGGDbqxcqGH9aqpcqWGTbqBcqGHPms4cuWGUbGBgaqcamaaBaaaleaacqWGQbGAaeqaaOGaeyOkJeVaey4kaSIaf8NSdiMbaKaaaaa@3B8D@, and [n^]=mn^j
 MathType@MTEF@5@5@+=feaafiart1ev1aaatCvAUfKttLearuWrP9MDH5MBPbIqV92AaeXatLxBI9gBaebbnrfifHhDYfgasaacPC6xNi=xH8viVGI8Gi=hEeeu0xXdbba9frFj0xb9qqpG0dXdb9aspeI8k8fiI+fsY=rqGqVepae9pg0db9vqaiVgFr0xfr=xfr=xc9adbaqaaeGacaGaaiaabeqaaeqabiWaaaGcbaGaei4waSLafmOBa4MbaKaacqGGDbqxcqGH9aqpcqWGTbqBcuWGUbGBgaqcamaaBaaaleaacqWGQbGAaeqaaaaa@352F@. Using this notation, a comparison of the definition of *η*^2 ^with (4) shows that we can write

η2(I^j)=η2([αn_])+η2([β^])=η2([n^])+σα2var⁡(n^jN^(0,1))/〈n^j〉2+η2([β^]).
 MathType@MTEF@5@5@+=feaafiart1ev1aaatCvAUfKttLearuWrP9MDH5MBPbIqV92AaeXatLxBI9gBaebbnrfifHhDYfgasaacPC6xNi=xI8qiVKYPFjYdHaVhbbf9v8qqaqFr0xc9vqFj0dXdbba91qpepeI8k8fiI+fsY=rqGqVepae9pg0db9vqaiVgFr0xfr=xfr=xc9adbaqaaeGacaGaaiaabeqaaeqabiWaaaGcbaqbaeaabiWaaaqaaGGaciab=D7aOnaaCaaaleqabaGaeGOmaidaaOGaeiikaGIafmysaKKbaKaadaWgaaWcbaGaemOAaOgabeaakiabcMcaPaqaaiabg2da9aqaaiab=D7aOnaaCaaaleqabaGaeGOmaidaaOGaeiikaGIaei4waS1aaecaaeaacqWFXoqycqWGUbGBaiaawkWaaiabc2faDjabcMcaPiabgUcaRiab=D7aOnaaCaaaleqabaGaeGOmaidaaOGaeiikaGIaei4waSLaf8NSdiMbaKaacqGGDbqxcqGGPaqkaeaaaeaacqGH9aqpaeaacqWF3oaAdaahaaWcbeqaaiabikdaYaaakiabcIcaOiabcUfaBjqbd6gaUzaajaGaeiyxa0LaeiykaKIaey4kaSIae83Wdm3aa0baaSqaaiab=f7aHbqaaiabikdaYaaakiGbcAha2jabcggaHjabckhaYjabcIcaOiqbd6gaUzaajaWaaSbaaSqaaiabdQgaQbqabaGccuWGobGtgaqcaiabcIcaOiabicdaWiabcYcaSiabigdaXiabcMcaPiabcMcaPiabc+caViabgMYiHlqbd6gaUzaajaWaaSbaaSqaaiabdQgaQbqabaGccqGHQms8daahaaWcbeqaaiabikdaYaaakiabgUcaRiab=D7aOnaaCaaaleqabaGaeGOmaidaaOGaeiikaGIaei4waSLaf8NSdiMbaKaacqGGDbqxcqGGPaqkcqGGUaGlaaaaaa@7951@

The middle term of the above equation simplifies even further when the molecular number is large so that n^j=〈n^j〉
 MathType@MTEF@5@5@+=feaafiart1ev1aaatCvAUfKttLearuWrP9MDH5MBPbIqV92AaeXatLxBI9gBaebbnrfifHhDYfgasaacPC6xNi=xH8viVGI8Gi=hEeeu0xXdbba9frFj0xb9qqpG0dXdb9aspeI8k8fiI+fsY=rqGqVepae9pg0db9vqaiVgFr0xfr=xfr=xc9adbaqaaeGacaGaaiaabeqaaeqabiWaaaGcbaGafmOBa4MbaKaadaWgaaWcbaGaemOAaOgabeaakiabg2da9iabgMYiHlqbd6gaUzaajaWaaSbaaSqaaiabdQgaQbqabaGccqGHQms8aaa@366C@ and hence σα2var⁡(n^jN^(0,1))=σα2
 MathType@MTEF@5@5@+=feaafiart1ev1aaatCvAUfKttLearuWrP9MDH5MBPbIqV92AaeXatLxBI9gBaebbnrfifHhDYfgasaacPC6xNi=xH8viVGI8Gi=hEeeu0xXdbba9frFj0xb9qqpG0dXdb9aspeI8k8fiI+fsY=rqGqVepae9pg0db9vqaiVgFr0xfr=xfr=xc9adbaqaaeGacaGaaiaabeqaaeqabiWaaaGcbaacciGae83Wdm3aa0baaSqaaiab=f7aHbqaaiabikdaYaaakiGbcAha2jabcggaHjabckhaYjabcIcaOiqbd6gaUzaajaWaaSbaaSqaaiabdQgaQbqabaGccuWGobGtgaqcaiabcIcaOiabicdaWiabcYcaSiabigdaXiabcMcaPiabcMcaPiabg2da9iab=n8aZnaaDaaaleaacqWFXoqyaeaacqaIYaGmaaaaaa@4468@. If the contribution of background noise β^
 MathType@MTEF@5@5@+=feaafiart1ev1aaatCvAUfKttLearuWrP9MDH5MBPbIqV92AaeXatLxBI9gBaebbnrfifHhDYfgasaacPC6xNi=xH8viVGI8Gi=hEeeu0xXdbba9frFj0xb9qqpG0dXdb9aspeI8k8fiI+fsY=rqGqVepae9pg0db9vqaiVgFr0xfr=xfr=xc9adbaqaaeGacaGaaiaabeqaaeqabiWaaaGcbaacciGaf8NSdiMbaKaaaaa@2D8B@ is also small then it is the case that

η2(I^j)=σα2=η2([α^]).
 MathType@MTEF@5@5@+=feaafiart1ev1aaatCvAUfKttLearuWrP9MDH5MBPbIqV92AaeXatLxBI9gBaebbnrfifHhDYfgasaacPC6xNi=xI8qiVKYPFjYdHaVhbbf9v8qqaqFr0xc9vqFj0dXdbba91qpepeI8k8fiI+fsY=rqGqVepae9pg0db9vqaiVgFr0xfr=xfr=xc9adbaqaaeGacaGaaiaabeqaaeqabiWaaaGcbaacciGae83TdG2aaWbaaSqabeaacqaIYaGmaaGccqGGOaakcuWGjbqsgaqcamaaBaaaleaacqWGQbGAaeqaaOGaeiykaKIaeyypa0Jae83Wdm3aa0baaSqaaiab=f7aHbqaaiabikdaYaaakiabg2da9iab=D7aOnaaCaaaleqabaGaeGOmaidaaOGaeiikaGIaei4waSLaf8xSdeMbaKaacqGGDbqxcqGGPaqkcqGGUaGlaaa@438A@

In summary, when Bcd molecular number is large in the anterior region of the embryo, we expect to see a constant level of normalized variance in our fluorescence data, contributed solely by rescaling noise α^
 MathType@MTEF@5@5@+=feaafiart1ev1aaatCvAUfKttLearuWrP9MDH5MBPbIqV92AaeXatLxBI9gBaebbnrfifHhDYfgasaacPC6xNi=xH8viVGI8Gi=hEeeu0xXdbba9frFj0xb9qqpG0dXdb9aspeI8k8fiI+fsY=rqGqVepae9pg0db9vqaiVgFr0xfr=xfr=xc9adbaqaaeGacaGaaiaabeqaaeqabiWaaaGcbaacciGaf8xSdeMbaKaaaaa@2D89@. Thus, α^
 MathType@MTEF@5@5@+=feaafiart1ev1aaatCvAUfKttLearuWrP9MDH5MBPbIqV92AaeXatLxBI9gBaebbnrfifHhDYfgasaacPC6xNi=xH8viVGI8Gi=hEeeu0xXdbba9frFj0xb9qqpG0dXdb9aspeI8k8fiI+fsY=rqGqVepae9pg0db9vqaiVgFr0xfr=xfr=xc9adbaqaaeGacaGaaiaabeqaaeqabiWaaaGcbaacciGaf8xSdeMbaKaaaaa@2D89@ can be identified independently from *m *and other random variables in our data.

### Physical constraints from a high-variance ensemble of embryos

**Figure 2:**
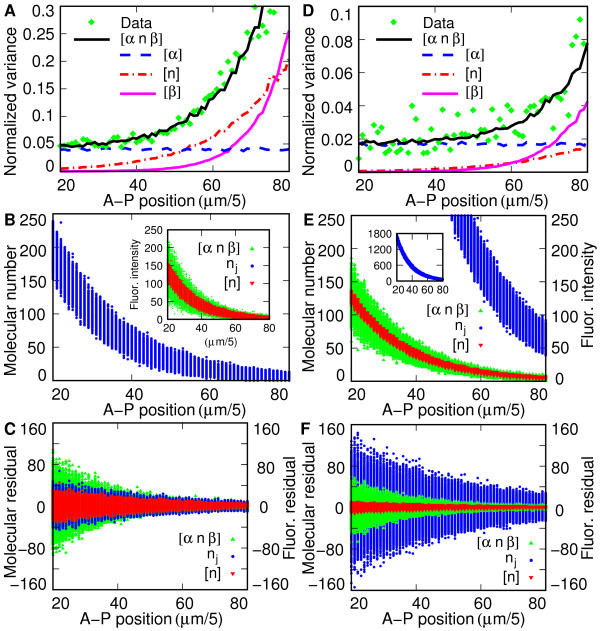
**Physical constraints of Bicoid gradient from the ensemble of embryos data**. (**A**) The normalized variance. In the key, "Data" denotes the members of η2(∪iIij)
 MathType@MTEF@5@5@+=feaafiart1ev1aaatCvAUfKttLearuWrP9MDH5MBPbIqV92AaeXatLxBI9gBaebbnrfifHhDYfgasaacPC6xNi=xH8viVGI8Gi=hEeeu0xXdbba9frFj0xb9qqpG0dXdb9aspeI8k8fiI+fsY=rqGqVepae9pg0db9vqaiVgFr0xfr=xfr=xc9adbaqaaeGacaGaaiaabeqaaeqabiWaaaGcbaacciGae83TdG2aaWbaaSqabeaacqaIYaGmaaGccqGGOaakdaWeqbqaaiabdMeajnaaBaaaleaacqWGPbqAcqWGQbGAaeqaaaqaaiabdMgaPbqab0GaeSOkIufakiabcMcaPaaa@376E@ from the high-variance ensemble of 89 embryos. Lines denote simulation results as shown in the key. [*αnβ*] denotes the full model η2(I^j)=η2([αnβ_])
 MathType@MTEF@5@5@+=feaafiart1ev1aaatCvAUfKttLearuWrP9MDH5MBPbIqV92AaeXatLxBI9gBaebbnrfifHhDYfgasaacPC6xNi=xH8viVGI8Gi=hEeeu0xXdbba9frFj0xb9qqpG0dXdb9aspeI8k8fiI+fsY=rqGqVepae9pg0db9vqaiVgFr0xfr=xfr=xc9adbaqaaeGacaGaaiaabeqaaeqabiWaaaGcbaacciGae83TdG2aaWbaaSqabeaacqaIYaGmaaGccqGGOaakcuWGjbqsgaqcamaaBaaaleaacqWGQbGAaeqaaOGaeiykaKIaeyypa0Jae83TdG2aaWbaaSqabeaacqaIYaGmaaGccqGGOaakcqGGBbWwdaqiaaqaaiab=f7aHjabd6gaUjab=j7aIbGaayPadaGaeiyxa0LaeiykaKcaaa@4084@, [*α*] denotes η2([α^])
 MathType@MTEF@5@5@+=feaafiart1ev1aaatCvAUfKttLearuWrP9MDH5MBPbIqV92AaeXatLxBI9gBaebbnrfifHhDYfgasaacPC6xNi=xH8viVGI8Gi=hEeeu0xXdbba9frFj0xb9qqpG0dXdb9aspeI8k8fiI+fsY=rqGqVepae9pg0db9vqaiVgFr0xfr=xfr=xc9adbaqaaeGacaGaaiaabeqaaeqabiWaaaGcbaacciGae83TdG2aaWbaaSqabeaacqaIYaGmaaGccqGGOaakcqGGBbWwcuWFXoqygaqcaiabc2faDjabcMcaPaaa@348B@, [*n*] denotes η2([n^])
 MathType@MTEF@5@5@+=feaafiart1ev1aaatCvAUfKttLearuWrP9MDH5MBPbIqV92AaeXatLxBI9gBaebbnrfifHhDYfgasaacPC6xNi=xH8viVGI8Gi=hEeeu0xXdbba9frFj0xb9qqpG0dXdb9aspeI8k8fiI+fsY=rqGqVepae9pg0db9vqaiVgFr0xfr=xfr=xc9adbaqaaeGacaGaaiaabeqaaeqabiWaaaGcbaacciGae83TdG2aaWbaaSqabeaacqaIYaGmaaGccqGGOaakcqGGBbWwcuWGUbGBgaqcaiabc2faDjabcMcaPaaa@3456@, and [*β*] denotes η2([β^])
 MathType@MTEF@5@5@+=feaafiart1ev1aaatCvAUfKttLearuWrP9MDH5MBPbIqV92AaeXatLxBI9gBaebbnrfifHhDYfgasaacPC6xNi=xH8viVGI8Gi=hEeeu0xXdbba9frFj0xb9qqpG0dXdb9aspeI8k8fiI+fsY=rqGqVepae9pg0db9vqaiVgFr0xfr=xfr=xc9adbaqaaeGacaGaaiaabeqaaeqabiWaaaGcbaacciGae83TdG2aaWbaaSqabeaacqaIYaGmaaGccqGGOaakcqGGBbWwcuWFYoGygaqcaiabc2faDjabcMcaPaaa@348D@. The parameters used in simulation were *σ*_*α *_= 0.2, *σ*_*β *_= 1.7, *J *= 30 (molecules/s), *m *= 0.7, *D *= 17.2 (*μ*m^2^/s) and *ω *= 0.0027 (s^-1^). (**B**) is a scatterplot of Bcd molecular number n^j
 MathType@MTEF@5@5@+=feaafiart1ev1aaatCvAUfKttLearuWrP9MDH5MBPbIqV92AaeXatLxBI9gBaebbnrfifHhDYfgasaacPC6xNi=xH8viVGI8Gi=hEeeu0xXdbba9frFj0xb9qqpG0dXdb9aspeI8k8fiI+fsY=rqGqVepae9pg0db9vqaiVgFr0xfr=xfr=xc9adbaqaaeGacaGaaiaabeqaaeqabiWaaaGcbaGafmOBa4MbaKaadaWgaaWcbaGaemOAaOgabeaaaaa@2ED1@, while the inset shows simulated fluorescence intensity for models and parameters used in panel A. (**C**) shows the residuals (deviations from mean) of panel B. (**D**), (**E**) and (**F**) show the same information as panel A, B, and C respectively, but from the low-variance ensemble of 17 embryos, with parameters used in simulation *σ*_*α *_= 0.13, *σ*_*β*_, = 1.0, *J *= 200 (molecules/s), *m *= 0.07, *D *= 17.2 (*μ*m^2^/s) and *ω *= 0.00215 (s^-1^). Note that the axes in panel A and D are scaled differently, and the absolute molecule number is shown in the inset to panel E.

We first show results of the above statistical comparison using all 89 FlyEx cycle 13 embryos. This ensemble contains 9400 nuclei, with about 150 nuclei per bin. In Figure 2A we see that the normalized variance of the ensemble data η2(∪iIij)
 MathType@MTEF@5@5@+=feaafiart1ev1aaatCvAUfKttLearuWrP9MDH5MBPbIqV92AaeXatLxBI9gBaebbnrfifHhDYfgasaacPC6xNi=xH8viVGI8Gi=hEeeu0xXdbba9frFj0xb9qqpG0dXdb9aspeI8k8fiI+fsY=rqGqVepae9pg0db9vqaiVgFr0xfr=xfr=xc9adbaqaaeGacaGaaiaabeqaaeqabiWaaaGcbaacciGae83TdG2aaWbaaSqabeaacqaIYaGmaaGccqGGOaakdaWeqbqaaiabdMeajnaaBaaaleaacqWGPbqAcqWGQbGAaeqaaaqaaiabdMgaPbqab0GaeSOkIufakiabcMcaPaaa@376E@ asymptotically approaches the simulation curve η2([α^])=σα2
 MathType@MTEF@5@5@+=feaafiart1ev1aaatCvAUfKttLearuWrP9MDH5MBPbIqV92AaeXatLxBI9gBaebbnrfifHhDYfgasaacPC6xNi=xH8viVGI8Gi=hEeeu0xXdbba9frFj0xb9qqpG0dXdb9aspeI8k8fiI+fsY=rqGqVepae9pg0db9vqaiVgFr0xfr=xfr=xc9adbaqaaeGacaGaaiaabeqaaeqabiWaaaGcbaacciGae83TdG2aaWbaaSqabeaacqaIYaGmaaGccqGGOaakcqGGBbWwcuWFXoqygaqcaiabc2faDjabcMcaPiabg2da9iab=n8aZnaaDaaaleaacqWFXoqyaeaacqaIYaGmaaaaaa@3A08@ in the anterior region of the embryo. *σ*_*α *_values that are too high will place the black model curve above the data points on the left, and this constrains *σ*_*α *_to be less than 0.2.

With regard to the molecular parameters of diffusion, adopting the value of *D *= 17.2 (*μ*m^2^/s) given in the literature [20] together with our observed value of *λ *= 80.65 (*μ*m) for this ensemble implies that *ω *= 0.0027 (s^-1^) in order to satisfy (8a). These values, for any *J*, will cause the mean molecular number gradient, 〈n^j〉
 MathType@MTEF@5@5@+=feaafiart1ev1aaatCvAUfKttLearuWrP9MDH5MBPbIqV92AaeXatLxBI9gBaebbnrfifHhDYfgasaacPC6xNi=xH8viVGI8Gi=hEeeu0xXdbba9frFj0xb9qqpG0dXdb9aspeI8k8fiI+fsY=rqGqVepae9pg0db9vqaiVgFr0xfr=xfr=xc9adbaqaaeGacaGaaiaabeqaaeqabiWaaaGcbaGaeyykJeUafmOBa4MbaKaadaWgaaWcbaGaemOAaOgabeaakiabgQYiXdaa@325E@, to be in steady state after about 4000 seconds (cycle 8). A lower bound of *J > *30 (molecules/s) and an upper bound of *m <*0.7 is imposed by the experimentally observed magnitude of *η*^2^. Finally, we estimate the constraint for background noise β^
 MathType@MTEF@5@5@+=feaafiart1ev1aaatCvAUfKttLearuWrP9MDH5MBPbIqV92AaeXatLxBI9gBaebbnrfifHhDYfgasaacPC6xNi=xH8viVGI8Gi=hEeeu0xXdbba9frFj0xb9qqpG0dXdb9aspeI8k8fiI+fsY=rqGqVepae9pg0db9vqaiVgFr0xfr=xfr=xc9adbaqaaeGacaGaaiaabeqaaeqabiWaaaGcbaacciGaf8NSdiMbaKaaaaa@2D8B@ by satisfying (8b) in the posterior end of the embryo. Violation of the inequality (8b) would cause the black model curve to be above the data on the right hand side of Figure 2A, and hence we have an upper bound *σ*_*β *_< 1.7.

In summary, analysis of our high-variance ensemble of embryos dataset implies that the Bcd synthesis rate is higher than 30 (molecules/s). Figure 2B shows a scatterplot of the molecular number associated with this synthesis rate. The panel indicates that most nuclei in the anterior fifth of the embryo contain more than 200 molecules of Bcd after reaching steady state, and that these molecular numbers can fluctuate over a range of more than 80 molecules in this region (Fig. 2C). This panel shows that these molecular fluctuations, even at the largest level of normalized variance compatible with data, still do not account for the observed variance in experimental observations. The additional variance comes chiefly from rescaling noise α^
 MathType@MTEF@5@5@+=feaafiart1ev1aaatCvAUfKttLearuWrP9MDH5MBPbIqV92AaeXatLxBI9gBaebbnrfifHhDYfgasaacPC6xNi=xH8viVGI8Gi=hEeeu0xXdbba9frFj0xb9qqpG0dXdb9aspeI8k8fiI+fsY=rqGqVepae9pg0db9vqaiVgFr0xfr=xfr=xc9adbaqaaeGacaGaaiaabeqaaeqabiWaaaGcbaacciGaf8xSdeMbaKaaaaa@2D89@. Background noise β^
 MathType@MTEF@5@5@+=feaafiart1ev1aaatCvAUfKttLearuWrP9MDH5MBPbIqV92AaeXatLxBI9gBaebbnrfifHhDYfgasaacPC6xNi=xH8viVGI8Gi=hEeeu0xXdbba9frFj0xb9qqpG0dXdb9aspeI8k8fiI+fsY=rqGqVepae9pg0db9vqaiVgFr0xfr=xfr=xc9adbaqaaeGacaGaaiaabeqaaeqabiWaaaGcbaacciGaf8NSdiMbaKaaaaa@2D8B@ only has a significant effect at the posterior end of the embryo, and indeed dominates the normalized variance in that region (Fig. 2A). Towards the posterior, η2([β^])
 MathType@MTEF@5@5@+=feaafiart1ev1aaatCvAUfKttLearuWrP9MDH5MBPbIqV92AaeXatLxBI9gBaebbnrfifHhDYfgasaacPC6xNi=xH8viVGI8Gi=hEeeu0xXdbba9frFj0xb9qqpG0dXdb9aspeI8k8fiI+fsY=rqGqVepae9pg0db9vqaiVgFr0xfr=xfr=xc9adbaqaaeGacaGaaiaabeqaaeqabiWaaaGcbaacciGae83TdG2aaWbaaSqabeaacqaIYaGmaaGccqGGOaakcqGGBbWwcuWFYoGygaqcaiabc2faDjabcMcaPaaa@348D@ rises faster than η2(n^j)
 MathType@MTEF@5@5@+=feaafiart1ev1aaatCvAUfKttLearuWrP9MDH5MBPbIqV92AaeXatLxBI9gBaebbnrfifHhDYfgasaacPC6xNi=xH8viVGI8Gi=hEeeu0xXdbba9frFj0xb9qqpG0dXdb9aspeI8k8fiI+fsY=rqGqVepae9pg0db9vqaiVgFr0xfr=xfr=xc9adbaqaaeGacaGaaiaabeqaaeqabiWaaaGcbaacciGae83TdG2aaWbaaSqabeaacqaIYaGmaaGccqGGOaakcuWGUbGBgaqcamaaBaaaleaacqWGQbGAaeqaaOGaeiykaKcaaa@3369@, and its sharp rise may in certain cases serve as a marker to distinguish regimes dominated by molecular noise from those dominated by background noise.

### Physical constraints from a low-variance ensemble of embryos

In the ensemble of embryos discussed above, a portion of the variance observed is likely to stem from embryo to embryo variation in staining conditions and inherent biological parameters. In order to find a better upper limit for observed molecular fluctuations, it is desirable to analyze a set of embryos whose properties are as uniform as possible. In order to generate such a set, we considered the value of *λ*_*i *_in the exponential fit F [*I*_*ij*_] = *a*_*i *_exp(-*j*/*λ*_*i*_) to each embryo. We then grouped embryos according to common values of 1/*λ*_*i*_, taken to two decimal places. In the largest such group, which contained 17 members, we rescaled the data to a common amplitude by letting

I′ij=〈ai〉aiIij.
 MathType@MTEF@5@5@+=feaafiart1ev1aaatCvAUfKttLearuWrP9MDH5MBPbIqV92AaeXatLxBI9gBaebbnrfifHhDYfgasaacPC6xNi=xI8qiVKYPFjYdHaVhbbf9v8qqaqFr0xc9vqFj0dXdbba91qpepeI8k8fiI+fsY=rqGqVepae9pg0db9vqaiVgFr0xfr=xfr=xc9adbaqaaeGacaGaaiaabeqaaeqabiWaaaGcbaqcfaOafmysaKKbauaadaWgaaqaaiabdMgaPjabdQgaQbqabaGaeyypa0ZaaSaaaeaacqGHPms4cqWGHbqydaWgaaqaaiabdMgaPbqabaGaeyOkJepabaGaemyyae2aaSbaaeaacqWGPbqAaeqaaaaacqWGjbqsdaWgaaqaaiabdMgaPjabdQgaQbqabaGaeiOla4caaa@3FAE@

These 17 processed embryos constitute an ensemble ∪iI′ij
 MathType@MTEF@5@5@+=feaafiart1ev1aaatCvAUfKttLearuWrP9MDH5MBPbIqV92AaeXatLxBI9gBaebbnrfifHhDYfgasaacPC6xNi=xH8viVGI8Gi=hEeeu0xXdbba9frFj0xb9qqpG0dXdb9aspeI8k8fiI+fsY=rqGqVepae9pg0db9vqaiVgFr0xfr=xfr=xc9adbaqaaeGacaGaaiaabeqaaeqabiWaaaGcbaWaambuaeaacuWGjbqsgaqbamaaBaaaleaacqWGPbqAcqWGQbGAaeqaaaqaaiabdMgaPbqab0GaeSOkIufaaaa@32E2@ for statistical analysis as described in the previous section. A trade-off of this treatment is the loss of statistical sample size, with only around 30 nuclei in each bin.

Figure 2D shows that this ensemble of 17 embryos has lower normalized variance compared to the 89 embryos ensemble in Figure 2A. The fluctuation of normalized variance is also higher because of smaller sample size. Note that rescaling noise is dominant over a larger portion of the embryo than is the case for the full 89 embryo ensemble. We estimate an upper bound for rescaling noise *σ*_*α *_to be 0.13. At this point it is possible to determine the smallest *J *compatible with variance as was done for the high variance ensemble. We do not do so, however, because in this ensemble the contribution of β^
 MathType@MTEF@5@5@+=feaafiart1ev1aaatCvAUfKttLearuWrP9MDH5MBPbIqV92AaeXatLxBI9gBaebbnrfifHhDYfgasaacPC6xNi=xH8viVGI8Gi=hEeeu0xXdbba9frFj0xb9qqpG0dXdb9aspeI8k8fiI+fsY=rqGqVepae9pg0db9vqaiVgFr0xfr=xfr=xc9adbaqaaeGacaGaaiaabeqaaeqabiWaaaGcbaacciGaf8NSdiMbaKaaaaa@2D8B@ is too small to be separated from n^j
 MathType@MTEF@5@5@+=feaafiart1ev1aaatCvAUfKttLearuWrP9MDH5MBPbIqV92AaeXatLxBI9gBaebbnrfifHhDYfgasaacPC6xNi=xH8viVGI8Gi=hEeeu0xXdbba9frFj0xb9qqpG0dXdb9aspeI8k8fiI+fsY=rqGqVepae9pg0db9vqaiVgFr0xfr=xfr=xc9adbaqaaeGacaGaaiaabeqaaeqabiWaaaGcbaGafmOBa4MbaKaadaWgaaWcbaGaemOAaOgabeaaaaa@2ED1@.

We compared this data to simulations performed using the same parameter values reported in the previous section, except that *λ *(for the whole ensemble) had a value of 90.91 (*μ*m), leading to *ω *taking on a value of 0.00215 (s^-1^). These values cause the deterministic system to relax to steady state after 4000 seconds, as was the case with the high variance ensemble. In the high variance ensemble (Fig. 2A), the choice of *σ*_*β *_was dictated by the necessity of matching the observed variance along the entire A-P axis. In the case of the low variance ensemble this is not required, but measurements from nonexpressing nuclei indicate that *σ*_*β *_is equal to about 1. It is important to have at least a rough estimate for *σ*_*β *_so that fluctuations from this source are not spuriously assigned to fluctuations in molecular number. Finally, these constraints require that the lower bound of synthesis rate *J *be 200 (molecules/s) in order that that η2(I^j)≃η2(∪iI′ij)
 MathType@MTEF@5@5@+=feaafiart1ev1aaatCvAUfKttLearuWrP9MDH5MBPbIqV92AaeXatLxBI9gBaebbnrfifHhDYfgasaacPC6xNi=xH8viVGI8Gi=hEeeu0xXdbba9frFj0xb9qqpG0dXdb9aspeI8k8fiI+fsY=rqGqVepae9pg0db9vqaiVgFr0xfr=xfr=xc9adbaqaaeGacaGaaiaabeqaaeqabiWaaaGcbaacciGae83TdG2aaWbaaSqabeaacqaIYaGmaaGccqGGOaakcuWGjbqsgaqcamaaBaaaleaacqWGQbGAaeqaaOGaeiykaKIaeS4qISJae83TdG2aaWbaaSqabeaacqaIYaGmaaGccqGGOaakdaWeqbqaaiqbdMeajzaafaWaaSbaaSqaaiabdMgaPjabdQgaQbqabaaabaGaemyAaKgabeqdcqWIQisvaOGaeiykaKcaaa@3FEB@. The corresponding upper bound of molecule-to-fluorescence rescaling ratio is *m *= 0.07.

The lower limit of *J *imposed by this ensemble of low variance embryos in turn implies that there must be more than 300 Bcd molecules per subvolume in the middle of the embryo (*j *= 50) after reaching steady state (Fig. 2E). Moreover, it implies that the Bcd molecular gradient does not drop to 0 as the fluorescence intensity reaches the presumed background level, but remains at a level of at least 50 molecules per subvolume at *j *= 80, and 36 molecules per subvolume at the posterior pole (*j *= 100). Note that the variance of fluorescence measurements is similar between the two embryo ensembles (compare the green areas in Fig. 2C and Fig. 2F), but that the portion of that variance assigned to fluctuations in molecular number is smaller for the ensemble of 17 embryos (compare the red areas in Fig. 2C and Fig. 2F). The tighter constraints from the smaller ensemble make the lower limits on molecular number higher (compare Fig. 2B and Fig. 2E), and the lower limit on the variance of molecular number higher (compare the blue areas in Fig. 2C and Fig. 2F), although the higher limit of the normalized variance becomes smaller (compare the data points in Fig. 2A and Fig. 2D). The Bcd molecular number will thus vary by more than 100 molecules in the middle of the embryo. The 13% rescaling noise α^
 MathType@MTEF@5@5@+=feaafiart1ev1aaatCvAUfKttLearuWrP9MDH5MBPbIqV92AaeXatLxBI9gBaebbnrfifHhDYfgasaacPC6xNi=xH8viVGI8Gi=hEeeu0xXdbba9frFj0xb9qqpG0dXdb9aspeI8k8fiI+fsY=rqGqVepae9pg0db9vqaiVgFr0xfr=xfr=xc9adbaqaaeGacaGaaiaabeqaaeqabiWaaaGcbaacciGaf8xSdeMbaKaaaaa@2D89@ is still the main source of the characteristic variation observed in the anterior region of our fluorescence intensity data.

### Noise strength

**Figure 3:**
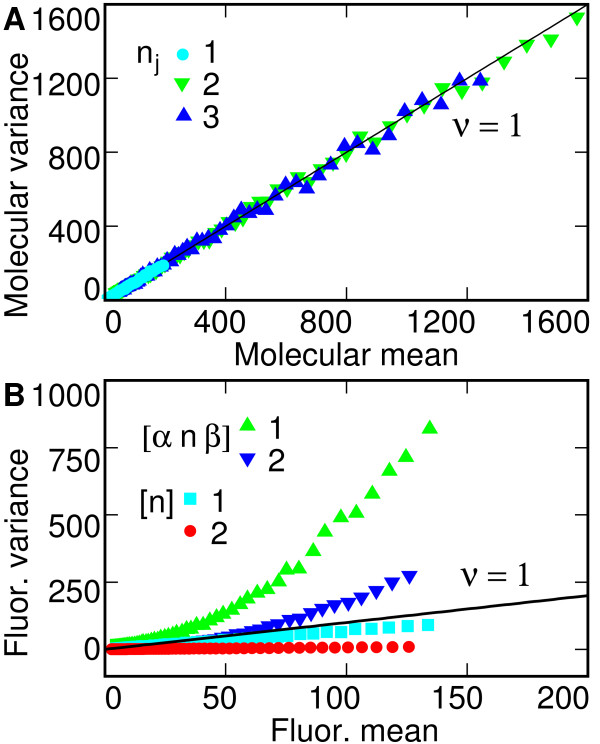
**Mean to variance correlation**. (**A**) The noise strength (Fano factor) *ν *of simulated Bicoid molecular number gradient is defined as molecular variance var⁡(n^j)
 MathType@MTEF@5@5@+=feaafiart1ev1aaatCvAUfKttLearuWrP9MDH5MBPbIqV92AaeXatLxBI9gBaebbnrfifHhDYfgasaacPC6xNi=xH8viVGI8Gi=hEeeu0xXdbba9frFj0xb9qqpG0dXdb9aspeI8k8fiI+fsY=rqGqVepae9pg0db9vqaiVgFr0xfr=xfr=xc9adbaqaaeGacaGaaiaabeqaaeqabiWaaaGcbaGagiODayNaeiyyaeMaeiOCaiNaeiikaGIafmOBa4MbaKaadaWgaaWcbaGaemOAaOgabeaakiabcMcaPaaa@34B9@ divided by molecular mean 〈n^j〉
 MathType@MTEF@5@5@+=feaafiart1ev1aaatCvAUfKttLearuWrP9MDH5MBPbIqV92AaeXatLxBI9gBaebbnrfifHhDYfgasaacPC6xNi=xH8viVGI8Gi=hEeeu0xXdbba9frFj0xb9qqpG0dXdb9aspeI8k8fiI+fsY=rqGqVepae9pg0db9vqaiVgFr0xfr=xfr=xc9adbaqaaeGacaGaaiaabeqaaeqabiWaaaGcbaGaeyykJeUafmOBa4MbaKaadaWgaaWcbaGaemOAaOgabeaakiabgQYiXdaa@325E@. The key indicates that parameters were obtained from (1) the high-variance ensemble of 89 embryos, (2) the low-variance ensemble of 17 embryos, and (3) the extreme condition of high diffusion rate *D *= 7890 (*μ*m^2^/s), decay rate *ω *= 1.0 (s^-1^) and synthesis rate *J *= 70000 (molecules/s). (**B**) As shown in the key, noise strength of simulated fluorescence intensity I^j=[αnβ_]
 MathType@MTEF@5@5@+=feaafiart1ev1aaatCvAUfKttLearuWrP9MDH5MBPbIqV92AaeXatLxBI9gBaebbnrfifHhDYfgasaacPC6xNi=xH8viVGI8Gi=hEeeu0xXdbba9frFj0xb9qqpG0dXdb9aspeI8k8fiI+fsY=rqGqVepae9pg0db9vqaiVgFr0xfr=xfr=xc9adbaqaaeGacaGaaiaabeqaaeqabiWaaaGcbaGafmysaKKbaKaadaWgaaWcbaGaemOAaOgabeaakiabg2da9iabcUfaBnaaHaaabaacciGae8xSdeMaemOBa4Mae8NSdigacaGLcmaacqGGDbqxaaa@3780@ and rescaled gradient [n^]
 MathType@MTEF@5@5@+=feaafiart1ev1aaatCvAUfKttLearuWrP9MDH5MBPbIqV92AaeXatLxBI9gBaebbnrfifHhDYfgasaacPC6xNi=xH8viVGI8Gi=hEeeu0xXdbba9frFj0xb9qqpG0dXdb9aspeI8k8fiI+fsY=rqGqVepae9pg0db9vqaiVgFr0xfr=xfr=xc9adbaqaaeGacaGaaiaabeqaaeqabiWaaaGcbaGaei4waSLafmOBa4MbaKaacqGGDbqxaaa@2FC8@ were obtained using parameters from (1) the high-variance ensemble of 89 embryos and (2) the low-variance ensemble of 17 embryos.

In most applications the most important measure of fluctuation is the normalized variance *η*^2 ^[21,22]. A different quantity, known as the Fano factor or noise strength [23-25], has been used by some authors as a marker to distinguish different stochastic mechanisms. The Fano factor *ν *is given by ν(I^j)=var⁡(I^j)/〈I^j〉
 MathType@MTEF@5@5@+=feaafiart1ev1aaatCvAUfKttLearuWrP9MDH5MBPbIqV92AaeXatLxBI9gBaebbnrfifHhDYfgasaacPC6xNi=xH8viVGI8Gi=hEeeu0xXdbba9frFj0xb9qqpG0dXdb9aspeI8k8fiI+fsY=rqGqVepae9pg0db9vqaiVgFr0xfr=xfr=xc9adbaqaaeGacaGaaiaabeqaaeqabiWaaaGcbaacciGae8xVd4MaeiikaGIafmysaKKbaKaadaWgaaWcbaGaemOAaOgabeaakiabcMcaPiabg2da9iGbcAha2jabcggaHjabckhaYjabcIcaOiqbdMeajzaajaWaaSbaaSqaaiabdQgaQbqabaGccqGGPaqkcqGGVaWlcqGHPms4cuWGjbqsgaqcamaaBaaaleaacqWGQbGAaeqaaOGaeyOkJepaaa@42CB@, where *ν *= 1 in a Poisson process. All stochastic simulations of Bcd intrinsic noise n^j
 MathType@MTEF@5@5@+=feaafiart1ev1aaatCvAUfKttLearuWrP9MDH5MBPbIqV92AaeXatLxBI9gBaebbnrfifHhDYfgasaacPC6xNi=xH8viVGI8Gi=hEeeu0xXdbba9frFj0xb9qqpG0dXdb9aspeI8k8fiI+fsY=rqGqVepae9pg0db9vqaiVgFr0xfr=xfr=xc9adbaqaaeGacaGaaiaabeqaaeqabiWaaaGcbaGafmOBa4MbaKaadaWgaaWcbaGaemOAaOgabeaaaaa@2ED1@ give *ν *= 1, as shown in Figure 3A. By contrast, the full statistical model for either of the two ensembles of embryos examined (green and blue data in Fig. 3B) is obviously non-Poisson, not only because *ν *≠ 1 but also because in the data, *ν *is a function of the mean. This happens because at larger values of molecular number, the variance of α^
 MathType@MTEF@5@5@+=feaafiart1ev1aaatCvAUfKttLearuWrP9MDH5MBPbIqV92AaeXatLxBI9gBaebbnrfifHhDYfgasaacPC6xNi=xH8viVGI8Gi=hEeeu0xXdbba9frFj0xb9qqpG0dXdb9aspeI8k8fiI+fsY=rqGqVepae9pg0db9vqaiVgFr0xfr=xfr=xc9adbaqaaeGacaGaaiaabeqaaeqabiWaaaGcbaacciGaf8xSdeMbaKaaaaa@2D89@ has a dominant role, even when its value is small. Even if we model our data without rescaling noise using the [n^]
 MathType@MTEF@5@5@+=feaafiart1ev1aaatCvAUfKttLearuWrP9MDH5MBPbIqV92AaeXatLxBI9gBaebbnrfifHhDYfgasaacPC6xNi=xH8viVGI8Gi=hEeeu0xXdbba9frFj0xb9qqpG0dXdb9aspeI8k8fiI+fsY=rqGqVepae9pg0db9vqaiVgFr0xfr=xfr=xc9adbaqaaeGacaGaaiaabeqaaeqabiWaaaGcbaGaei4waSLafmOBa4MbaKaacqGGDbqxaaa@2FC8@ random variable alone, uncertainty in the value of the rescaling constant *m *itself leads to ambiguity in the observed value of the Fano factor (red and cyan data in Fig. 3B). This is a natural consequence of the dimensions of the Fano factor.

## Conclusion

We have compared the nucleus to nucleus variation in expression levels of the exponentially distributed Bcd gradient observed in fixed tissue in a steady state with a stochastic model of the diffusion equation. The model is well supported, in the sense that there is a well-supported physical model for the spatial dependence of mean concentrations of Bcd [12,20] on the scale of the embryo. The first major result of our analysis is to note that in many individual embryos the nucleus to nucleus variation in the log of concentration is independent of spatial position. This pattern of variation, which amounts to multiplicative noise in concentration space, is completely incompatible with the stochastic behavior of the diffusion equation. Simulations of the diffusion equation over an exhaustively large region of parameter space without exception give rise to solutions in which nucleus to nucleus variation of the *bcd *gradient is a function of position in the embryo, whether this variation is measured directly in Bcd levels or in their logarithms.

The data which we compare the model to is in the form of fluorescence levels, not concentrations. Although there is now good evidence that the specific batch of serum used to obtain this data has a mean response to Bcd [26] which is linear, there is no quantitative information about the variance of this sensitivity. Previous work on intrinsic molecular noise in yeast and bacteria utilized GFP [27,28]*in vivo*, a situation where fluorescence is detected without molecular amplification. In the data reported here, and in most studies with fixed tissue, the signal from bound primary antibodies is amplified by incubation with secondary antibodies conjugated to a fluorescent dye. It is easily imaginable that this amplification process itself is subject to molecular fluctuations. These fluctuations would then give rise to rescaling noise in the proportionality between fluorescence levels and primary antigen molecular number from nucleus to nucleus. We have shown that such variation can explain the multiplicative noise observed.

When data from fixed embryos are pooled, better statistics are obtained. We analyzed data pooled from the entire dataset (*N *= 89) as well as a smaller pool of data from a set of embryos selected to have nearly identical mean Bcd profiles (*N *= 17). In order to analyze these data, we constructed an explicit statistical model. The model considers three sources of variance: intrinsic noise, rescaling noise, and background noise. By means of this statistical model it is possible to separate, at least roughly, the contributions of different sources of fluctuation. We have discussed mechanisms for the first two of these; the third, background noise, represents small fluctuations in the quantity of nonspecific molecules (background) from nucleus to nucleus. Because mean background has been previously removed from this data [29], the background noise has a mean of zero. We have confirmed that the background removal method does not affect our results, and the mean background intensity before removal is independent from the Bcd molecule-to-fluorescence rescaling ratio (data not shown). We also found from non-expressing areas of our data that the background noise (standard deviation) has about 54% positive correlation to the mean background intensity.

The results of this analysis constrained the physical parameters of the stochastic model considerably, with sharper constraints provided by the smaller dataset. The data require that the synthesis rate, *J*, of Bcd from its pool of anteriorly deposited mRNA be greater than 200 (molecules/s). We chose the subvolumes of the model to have the same volume as a nucleus, and hence the constraint on *J *also implies that there are a mean of at least 300 molecules of Bcd per nucleus at the midpoint of the embryo, and mean levels of at least 36 molecules of Bcd per nucleus at the posterior pole. In terms of concentration, our results show that Bcd concentration at the midpoint of the embryo is greater than 4 nM. Recently, a direct *in vivo *measurement of Bcd concentration was performed and yielded a mid embryo Bcd concentration of 8 nM [30], fully compatible with our results.

Although we are able to extract a clear signature of intrinsic molecular noise from the data by means of the statistical model, we also showed that at least one quantity diagnostic for different stochastic mechanisms, the Fano factor, cannot be read out from the data. Although the Fano factor *v *= 1 in the simulations, the full statistical model gives rise to a Fano factor which is a function of the mean, and even if all noise is restricted to be intrinsic, the observed Fano factor depends on the scale for conversion from fluorescence to molecular number.

We believe that our results in general demonstrate that fixed material processed with secondary fluorophores is not well suited to studies of molecular fluctuations. This arises from three issues, which may be separable. Fixation obviously prevents repeated observations on the same cell. While that is clearly a limitation, it need not affect an investigation of a molecule whose mean values are in steady state. The other two issues concern amplification. GFP is intrinsically fluorescent, but antibodies must bind to antigen, a process that is in itself subject to intrinsic molecular fluctuations. In the present study, the level of molecular fluctuation is doubtless increased by the need to bind secondary antibodies conjugated to fluorophore to the already bound primary antibody. It is thus possible that better data from fixed tissue could be obtained by conjugating dye directly to the primary antibody. This may prove to be a useful measure in situations where constructing a GFP fusion that can functionally substitute for the native gene is difficult or impossible.

More generally, we suggest that it is important to know how precision and robustness of developmental control is achieved at the molecular number level throughout development. Indeed, there is little doubt that stochastic processes are important later in development. Adult *Drosophila *normally have four scutellar bristles. In mutants of the *scute *gene, the number of bristles varies between one and three [31], strongly suggesting a stochastic process. On a more theoretical level, it has been suggested that fluctuations can augment the operating capabilities of biological regulatory networks [32]. Our results indicate that *in vivo *monitoring of gene expression will be required to obtain high quality data on stochastic gene expression phenomena in eucaryotes. The central technical problem that must be solved to conduct such studies is the complete replacement of the endogenous gene with a fluorescently tagged functional version [30].

## Methods

### Stochastic simulations

The Bcd gradient was modeled in one dimension with 101 homogeneous cubic subvolumes indexed by *j *from 0 to 100. Each subvolume has sides of length *l *= 5 *μ*m and volume Δ = *l*^3^. These dimensions were chosen in light of those of actual *Drosophila *embryos, which are 500 *μ*m long. The subvolume dimensions are very close to those of blastoderm nuclei. In the first subvolume (*j *= 0), corresponding to the anterior pole of the embryo, we assume a zero-order synthesis reaction of Bcd molecules with constant rate *J *(molecules/s), representing the translation of a maternally deposited and localized stationary mRNA pool after egg deposition. The *j*th subvolume contains *n*_*j *_molecules of Bcd, which are the state variables of the model. We take initial conditions to be *n*_*j *_= 0 ∀ *j*. Diffusion of Bcd is modeled as a first-order elementary reaction for the exchange of molecules between neighboring subvolumes with rate constant *k *= *D/l*^2 ^seconds^-1^, where *D *is the effective Fickian diffusion constant. Dispersed degradation (decay) of Bcd is also modeled as a first-order reaction in all subvolumes with rate constant *ω *(seconds^-1^).

Thus, for subvolumes *j *= 0 to *j *= 100, the RDME is given by

dP({nj},t)dt=(E0−1−1)JP({nj},t)+∑j=0100[(Ej+1−1)ωnjP({nj},t)]+∑j=0100∑k∈{j±1}[(Ej+1Ek−1−1)(Dl2)njP({nj},t)],
 MathType@MTEF@5@5@+=feaafiart1ev1aaatCvAUfKttLearuWrP9MDH5MBPbIqV92AaeXatLxBI9gBaebbnrfifHhDYfgasaacPC6xNi=xI8qiVKYPFjYdHaVhbbf9v8qqaqFr0xc9vqFj0dXdbba91qpepeI8k8fiI+fsY=rqGqVepae9pg0db9vqaiVgFr0xfr=xfr=xc9adbaqaaeGacaGaaiaabeqaaeqabiWaaaGcbaqbaeaabmWaaaqaaKqbaoaalaaabaGaemizaqMaemiuaaLaeiikaGIaei4EaSNaemOBa42aaSbaaeaacqWGQbGAaeqaaiabc2ha9jabcYcaSiabdsha0jabcMcaPaqaaiabdsgaKjabdsha0baaaOqaaiabg2da9aqaaiabcIcaOmrr1ngBPrwtHrhAYaqeguuDJXwAKbstHrhAGq1DVbaceaGae8hHWx0aa0baaSqaaiabicdaWaqaaiabgkHiTiabigdaXaaakiabgkHiTiabigdaXiabcMcaPiabdQeakjabdcfaqjabcIcaOiabcUha7jabd6gaUnaaBaaaleaacqWGQbGAaeqaaOGaeiyFa0NaeiilaWIaemiDaqNaeiykaKcabaaabaGaey4kaScabaWaaabCaeaacqGGBbWwcqGGOaakcqWFecFrdaqhaaWcbaGaemOAaOgabaGaey4kaSIaeGymaedaaOGaeyOeI0IaeGymaeJaeiykaKccciGae4xYdCNaemOBa42aaSbaaSqaaiabdQgaQbqabaGccqWGqbaucqGGOaakcqGG7bWEcqWGUbGBdaWgaaWcbaGaemOAaOgabeaakiabc2ha9jabcYcaSiabdsha0jabcMcaPiabc2faDbWcbaGaemOAaOMaeyypa0JaeGimaadabaGaeGymaeJaeGimaaJaeGimaadaniabggHiLdaakeaaaeaacqGHRaWkaeaadaaeWbqaamaaqafabaGaei4waSLaeiikaGIae8hHWx0aa0baaSqaaiabdQgaQbqaaiabgUcaRiabigdaXaaakiab=ri8fnaaDaaaleaacqWGRbWAaeaacqGHsislcqaIXaqmaaGccqGHsislcqaIXaqmcqGGPaqkcqGGOaakjuaGdaWcaaqaaiabdseaebqaaiabdYgaSnaaCaaabeqaaiabikdaYaaaaaGaeiykaKIaemOBa42aaSbaaeaacqWGQbGAaeqaaiabdcfaqjabcIcaOiabcUha7jabd6gaUnaaBaaabaGaemOAaOgabeaacqGG9bqFcqGGSaalcqWG0baDcqGGPaqkcqGGDbqxcqGGSaalaSqaaiabdUgaRjabgIGiolabcUha7jabdQgaQjabgglaXkabigdaXiabc2ha9bqab0GaeyyeIuoaaSqaaiabdQgaQjabg2da9iabicdaWaqaaiabigdaXiabicdaWiabicdaWaqdcqGHris5aaaaaaa@BC34@

where *P*({*n*_*j*_}, *t*) is the joint probability of state vector {*n*_*j*_} = [*n*_0_,..., *n*_*j*_,..., *n*_100_]. The state operator, E
 MathType@MTEF@5@5@+=feaafiart1ev1aaatCvAUfKttLearuWrP9MDH5MBPbIqV92AaeXatLxBI9gBaebbnrfifHhDYfgasaacPC6xNi=xH8viVGI8Gi=hEeeu0xXdbba9frFj0xb9qqpG0dXdb9aspeI8k8fiI+fsY=rqGqVepae9pg0db9vqaiVgFr0xfr=xfr=xc9adbaqaaeGacaGaaiaabeqaaeqabiWaaaGcbaWefv3ySLgznfgDOjdaryqr1ngBPrginfgDObcv39gaiqaacqWFecFraaa@37B3@, is defined so that Ej±1f(...,nj,...)=f(...,nj±1,...)
 MathType@MTEF@5@5@+=feaafiart1ev1aaatCvAUfKttLearuWrP9MDH5MBPbIqV92AaeXatLxBI9gBaebbnrfifHhDYfgasaacPC6xNi=xH8viVGI8Gi=hEeeu0xXdbba9frFj0xb9qqpG0dXdb9aspeI8k8fiI+fsY=rqGqVepae9pg0db9vqaiVgFr0xfr=xfr=xc9adbaqaaeGacaGaaiaabeqaaeqabiWaaaGcbaWefv3ySLgznfgDOjdaryqr1ngBPrginfgDObcv39gaiqaacqWFecFrdaqhaaWcbaGaemOAaOgabaGaeyySaeRaeGymaedaaOGaemOzayMaeiikaGIaeiOla4IaeiOla4IaeiOla4IaeiilaWIaemOBa42aaSbaaSqaaiabdQgaQbqabaGccqGGSaalcqGGUaGlcqGGUaGlcqGGUaGlcqGGPaqkcqGH9aqpcqWGMbGzcqGGOaakcqGGUaGlcqGGUaGlcqGGUaGlcqGGSaalcqWGUbGBdaWgaaWcbaGaemOAaOgabeaakiabgglaXkabigdaXiabcYcaSiabc6caUiabc6caUiabc6caUiabcMcaPaaa@5A37@. Monte Carlo simulations of the behavior of this equation were obtained using the publicly available software MesoRD 0.2.0 [33]. MesoRD can automatically generate a stochastic or deterministic model from its input, and we make use of this feature in the work presented here. In the deterministic limit, mesoRD calculates the mean trajectory 〈n^j〉
 MathType@MTEF@5@5@+=feaafiart1ev1aaatCvAUfKttLearuWrP9MDH5MBPbIqV92AaeXatLxBI9gBaebbnrfifHhDYfgasaacPC6xNi=xH8viVGI8Gi=hEeeu0xXdbba9frFj0xb9qqpG0dXdb9aspeI8k8fiI+fsY=rqGqVepae9pg0db9vqaiVgFr0xfr=xfr=xc9adbaqaaeGacaGaaiaabeqaaeqabiWaaaGcbaGaeyykJeUafmOBa4MbaKaadaWgaaWcbaGaemOAaOgabeaakiabgQYiXdaa@325E@(*t*). By converting the initial values of the state variables into concentrations, mesoRD can then integrate the classical Reaction-Diffusion Rate Equation (RDRE) given by

∂C∂t=D∂2C∂x−ωC,
 MathType@MTEF@5@5@+=feaafiart1ev1aaatCvAUfKttLearuWrP9MDH5MBPbIqV92AaeXatLxBI9gBaebbnrfifHhDYfgasaacPC6xNi=xI8qiVKYPFjYdHaVhbbf9v8qqaqFr0xc9vqFj0dXdbba91qpepeI8k8fiI+fsY=rqGqVepae9pg0db9vqaiVgFr0xfr=xfr=xc9adbaqaaeGacaGaaiaabeqaaeqabiWaaaGcbaqcfa4aaSaaaeaacqGHciITcqWGdbWqaeaacqGHciITcqWG0baDaaGaeyypa0Jaemiraq0aaSaaaeaacqGHciITdaahaaqabeaacqaIYaGmaaGaem4qameabaGaeyOaIyRaemiEaGhaaiabgkHiTGGaciab=L8a3jabdoeadjabcYcaSaaa@3F4A@

where *C *is the concentration of Bcd. Boundary conditions are given by ∂_*x*_*C*|_*x *= 0 _= -*J*/Δ and ∂_*x*_*C*|_*x *= 500 _= 0. The steady-state solution can be well approximated by *C*(*x*) ≈ *a *exp(-*x*/*λ*), where *a *= (*J*/Δ)*λ *and λ=(D/ω)
 MathType@MTEF@5@5@+=feaafiart1ev1aaatCvAUfKttLearuWrP9MDH5MBPbIqV92AaeXatLxBI9gBaebbnrfifHhDYfgasaacPC6xNi=xH8viVGI8Gi=hEeeu0xXdbba9frFj0xb9qqpG0dXdb9aspeI8k8fiI+fsY=rqGqVepae9pg0db9vqaiVgFr0xfr=xfr=xc9adbaqaaeGacaGaaiaabeqaaeqabiWaaaGcbaacciGae83UdWMaeyypa0ZaaOaaaeaacqGGOaakcqWGebarcqGGVaWlcqWFjpWDcqGGPaqkaSqabaaaaa@3420@. MesoRD solves the deterministic equation by fixing the mesh size at the number of subvolumes chosen for the stochastic system. We used the built-in Euler solver with a stepsize of 0.001 second after verifying that these settings yielded stable and accurate solutions. Further analysis of simulations in comparison with quantitative immunostained data were performed in MATLAB.

### Quantitative data

We used Bcd protein expression data from cleavage cycle 13 [34] that were downloaded from the FlyEx database [9,35]. In FlyEx, confocal scans have been processed into tables containing average fluorescence levels in each nucleus [36]; these fluorescence levels are linearly proportional to Bcd concentration [26], and hence to molecular number. Data were taken from the central 10% strip along the A-P axis with their D-V coordinate suppressed, and normalized to remove the non-specific background [29]. The gradient is in a steady state at cycle 13 [19]. For quantitative analysis and comparison with the model, we pooled data into 5 *μ*m 1D intervals to compare with the 5 *μ*m subvolumes of the stochastic model. For certain purposes we considered a 17 member subset of embryos among which the inverse of the spatial exponential coefficient *λ *varied by less than 1%. This subset contained embryos ab15, cb2, ac1, hz8, ac6, iz4, cb19, ac2, cb31, iz15, ac16, ad18, ad31, hz30, be1, hz33 and as22 from FlyEx.

## Authors' contributions

All authors participated in the design of the study, and wrote the manuscript together. YF conducted the analysis of data and simulations, and made the figures. All authors read and approved the final manuscript.
